# Unveiling the Mechanisms of Photocatalytic CO_2_ Reduction Reaction in Semiconductor/Metal Complex Hybrid Photocatalyst

**DOI:** 10.1002/smsc.70407

**Published:** 2026-09-18

**Authors:** Dasom Kim, Gayoung Ham, Jonghwan Lee, Yeseul Jo, Jeongu You, Seung Kyu Min, Hyojung Cha, Youn Jeong Jang

**Affiliations:** ^1^ Department of Chemical Engineering Hanyang University Seoul Republic of Korea; ^2^ School of Energy Engineering Kyungpook National University Daegu Republic of Korea; ^3^ Department of Chemistry Ulsan National Institute of Science and Technology Ulsan Republic of Korea

**Keywords:** charge dynamics, CO_2_ reduction mechanism, photocatalytic CO_2_ reduction, semiconductor and metal complex hybrid photocatalyst

## Abstract

Hybrid catalysts combining semiconductors with molecular metal complexes have emerged as a promising strategy for efficient solar‐to‐chemical conversion under mild conditions; however, the mechanistic origin of their catalytic selectivity remains poorly understood. Here, we report a TiO_2_/[Co(bpy)_3_]^2+^ hybrid photocatalyst that exhibits significantly enhanced activity and selectivity for CO_2_‐to‐CO conversion compared with pristine TiO_2_. By combining electrochemical analysis, DFT, and TAS, we address consecutive elementary steps of the photocatalytic cycle: electrochemical analysis establishes the conditions for catalyst activation, DFT identifies the thermodynamic origin of CO_2_ coordination selectivity at the activated center, and TAS provides supporting evidence for interfacial electron transfer dynamics under photocatalytic conditions. Specifically, DFT calculations show that CO_2_ coordination is thermodynamically favored over proton adsorption, providing a basis for the suppressed hydrogen evolution. TAS observations are consistent with fast electron injection from TiO_2_ to the Co–bpy complex and the formation of a long‐lived transient species under CO_2_ conditions, supporting the involvement of stabilized reduced intermediates in the proposed catalytic pathway. This interplay between interfacial charge transfer and CO_2_‐specific intermediate formation is consistent with the high selectivity toward CO production. These findings provide mechanistic insight into the roles of the semiconductor and molecular catalyst in hybrid photocatalysis.

## Graphical abstract

In TiO_2_/[Co(bpy)_3_]^2+^ hybrid photocatalytic system, the synergistic interplay between rapid interfacial charge transfer and the stabilization of catalytic intermediates is elucidated, as supported by DFT calculation and transient absorption spectroscopy, and governs the high selectivity for CO_2_‐to‐CO conversion.

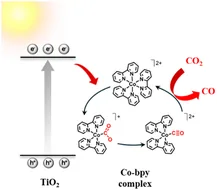

## 1 Introduction

The photocatalytic CO_2_ reduction reaction (CO_2_RR) has attracted considerable attention as a sustainable strategy for converting abundant solar energy into value‐added chemicals. Inorganic semiconductors, including TiO_2_ and CuInS_2_, have been extensively investigated as photocatalysts because their band structures provide sufficient thermodynamic driving force for CO_2_‐to‐CO conversion, enabling effective coupling with molecular catalysts [1, 2, 3]. However, their practical catalytic performance remains limited due to the rapid recombination of photogenerated charge carriers and the strong competition from the hydrogen evolution reaction (HER) [4]. These intrinsic limitations significantly suppress the CO production efficiency and selectivity. To address these challenges, hybrid photocatalytic systems that couple semiconductor light absorbers with molecular metal complexes have recently emerged as an effective strategy. In such systems, molecular catalysts dispersed in the reaction medium can serve as well‐defined catalytic centers, enabling more selective CO_2_ activation and conversion [5, 6, 7, 8, 9].

Molecular catalysts based on earth‐abundant metals such as Ni, Co, Mn, Fe, and Cu have been extensively investigated for CO_2_ reduction, typically coordinated with ligand frameworks including macrocycles, polypyridyl, porphyrins, and phosphines [10, 11, 12, 13, 14]. The catalytic performance of these complexes is largely governed by the electronic and steric properties of the metal center and ligand environment, which determine key parameters such as CO_2_ binding strength and the kinetics of proton‐coupled electron transfer [15, 16]. Despite significant progress in developing semiconductor–molecular complex hybrid catalysts, the fundamental understanding of their interfacial interactions remains incomplete [17, 18, 19, 20]. In particular, the mechanistic processes governing the generation, separation, and transfer of photoexcited charge carriers from the semiconductor to molecular catalytic sites—and their subsequent utilization in CO_2_ reduction—are still poorly understood. Addressing this knowledge gap is critical for the rational design of efficient hybrid photocatalytic systems.

To achieve a mechanistic understanding of CO_2_ conversion in hybrid photocatalytic systems, several key aspects are to be clarified. First, efficient transfer of photogenerated electrons from the semiconductor to the molecular metal complex is essential as this interfacial charge injection largely determines the efficiency of solar‐to‐chemical energy conversion. Second, the thermodynamically and kinetically favorable elementary steps governing the transformation of CO_2_ into CO must be identified. Most importantly, these photoinduced charge‐transfer processes and catalytic pathways should be established based on experimental observations under actual photocatalytic conditions, enabling a realistic interpretation of structure–activity relationships in hybrid photocatalysts.

In this work, we present a model TiO_2_/[Co(bpy)_3_]^2+^ hybrid photocatalyst for CO_2_‐to‐CO conversion. The hybrid catalyst exhibits pronounced CO production (16.52 μmol h^−1^) accompanied by minor H_2_ evolution (0.65 μmol h^−1^), representing a substantial improvement over pristine TiO_2_, which produces only 0.51 μmol h^−1^ of H_2_.

To elucidate the mechanisms governing photoexcited carrier generation, transport, and utilization during CO_2_ reduction, we combine electrochemical analysis, density functional theory (DFT), and transient absorption spectroscopy (TAS). DFT calculations show that CO_2_ coordination is thermodynamically favored over proton adsorption, accounting for the suppressed hydrogen evolution. Consistent with this, TAS reveals fast electron injection from TiO_2_ to the Co–bpy complex and a CO_2_‐specific, long‐lived transient species with concentration‐dependent kinetics, supporting the proposed mechanism. Therefore, these results demonstrate that the synergy between rapid interfacial charge transfer and stabilization of catalytic intermediates governs the high CO selectivity in CO_2_RR of the hybrid photocatalytic system. The combined experimental and theoretical investigations highlight the critical role of semiconductor–molecular catalyst coupling and provide mechanistic insight into the charge‐transfer processes and catalytic pathways that govern CO_2_ conversion. These findings offer important guidance for the rational design of advanced hybrid photocatalysts for solar‐driven chemical transformations.

## 2 Results and Discussion

### 2.1 Photocatalytic CO_2_ Reduction Using Hybrid Photocatalysts

In order to unveil the mechanisms of photocatalytic CO_2_RR in the semiconductor/metal complex, TiO_2_ and cobalt bipyridine, [Co(bpy)_3_]^2+^ (Co‐bpy), were chosen as a model hybrid photocatalyst. TiO_2_ presents a mixture of dominant anatase and rutile, as shown in Figure S1 [21]. Also, it shows uniformly spherical morphologies with an average diameter of 25 nm, as shown in Figure S2. A homogeneous catalyst of Co‐bpy is prepared by adding Co^2+^ and bpy in a reacting aqueous solution, enabling the self‐assembly of the Co–bpy complex. The UV–vis absorbance data in Figure S3 show the disappearance of the free CoCl_2_ absorption feature at 550–750 nm and the emergence of a new metal‐to‐ligand charge transfer band at 350–400 nm, supporting the formation of the Co–bpy complex [22].

**FIGURE 1:**
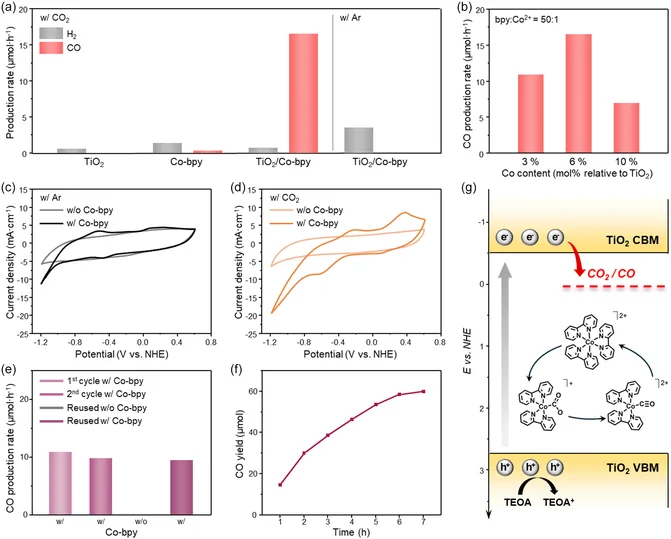
Photocatalytic CO_2_ reduction using hybrid photocatalysts. (a) H_2_ and CO production rates of pristine TiO_2_, Co‐bpy, and TiO_2_/Co‐bpy under CO_2_ or Ar atmosphere. (b) CO production rates as a function of Co^2+^ content (mol% relative to TiO_2_) at a fixed bpy/Co^2+^ ratio of 50:1. Cyclic voltammograms under (c) Ar and (d) CO_2_ saturation with and without Co‐bpy. (e) Photocatalytic CO_2_ reduction of the reused TiO_2_ with and without Co‐bpy. (f) Long‐term CO production of the optimized hybrid photocatalyst over 7 h. (g) Schematic illustration of the photoinduced charge transfer and selective CO_2_ reduction mechanism in the hybrid photocatalyst.

Under standard conditions in a CO_2_‐saturated mixed solution of acetonitrile/triethanolamine (TEOA)/H_2_O (4:1:1; v/v/v) under full‐spectrum illumination from an Hg lamp, the hybrid photocatalyst (6 mol% Co^2+^ relative to TiO_2_; bpy:Co ratio of 50:1) predominantly evolved CO (16.52 μmol h^−1^) with minor H_2_ evolution (0.65 μmol h^−1^) (Figure 1a and Table 1). This CO production rate far exceeded that of pristine TiO_2_, which exclusively generated H_2_ (0.51 μmol h^−1^). Interestingly, the major product was switched from H_2_ to CO when Co‐bpy was introduced under a CO_2_‐saturation. This distinct product shift indicates that the Co–bpy complex acts as a highly favorable active site for CO_2_ reduction compared to water reduction. While Co‐bpy alone could also drive CO_2_ reduction, its absolute gas production (0.23 μmol h^−1^ for CO and 1.44 μmol h^−1^ for H_2_) was drastically lower than that of the hybrid catalyst. This result clearly demonstrates the synergistic effect of the hybrid catalyst, where TiO_2_ functions as a light absorber and Co‐bpy serves as a catalytic center.

**TABLE 1:** Control experiments summarizing H_2_ and CO production rates for TiO_2_/Co‐bpy, pristine TiO_2_, and Co‐bpy under systematically varied conditions.

Catalyst	Light	Gas	Sacrificial h^+^ scavenger	H_2_ production rate, μmol h^−1^	CO production rate, μmol h^−1^
TiO_2_/Co‐bpy	Light (Hg lamp without cut‐off filter)	CO_2_	TEOA	0.65	16.52
				0.00	0.00
		Ar	TEOA	3.59	0.00
				0.00	0.00
	Dark	CO_2_	TEOA	0.00	0.00
TiO_2_	Light (Hg lamp without cut‐off filter)	CO_2_	TEOA	0.51	0.00
Co‐bpy				1.44	0.23

Control experiments further clarified the role of each component and origin of the CO source (Table 1). The absence of TEOA completely suppressed all gas production, confirming its essential role as a sacrificial hole scavenger. Most critically, replacing CO_2_ with Ar under otherwise identical conditions resulted in no detectable CO production, while H_2_ generation markedly increased (3.59 μmol h^−1^). Given that the hybrid catalyst exhibited a higher H_2_ production rate under Ar than pristine TiO_2_ (0.51 μmol h^−1^) or Co‐bpy (1.44 μmol h^−1^), it strategically demonstrates that even in the absence of CO_2_, the hybrid catalyst is exceptionally effective at harvesting photons and suppressing the recombination of photoexcited carriers, thereby maximizing the availability of reactive electrons for catalytic reduction. Also, this result suggests that light‐induced or photothermally assisted decomposition of Co‐bpy, TEOA, and acetonitrile is unlikely to contribute significantly to CO formation. Consistently, pristine TiO_2_ produced only H_2_ (0.51 umol h^−1^) but no detectable CO under CO_2_ in the presence of TEOA and acetonitrile, further arguing against these additives as carbonaceous contaminants. This interpretation was further supported by the ^13^CO_2_ isotope‐labeling experiment using differential electrochemical mass spectrometry (DEMS) (Figure S4).

To understand the factors influencing the hybrid photocatalysis, additional experiments were conducted. Stoichiometrically, the Co–bpy complex requires a 3:1 molar ratio of bpy‐to‐Co^2+^; however, the experimentally measured CO_2_RR activity increased progressively as the ratio was raised to 50:1, beyond which a marginal change was observed (Figure S5). Owing to the strong chelating affinity and established stepwise coordination equilibria of bpy, the bpy‐rich conditions may favor the formation and stabilization of highly coordinated Co‐bpy species, potentially including [Co(bpy)_3_]^2+^, in the initial reaction mixture [22, 23]. Also, free bpy may act as a ligand reservoir that supports recoordination of the ligand at the cobalt center following each catalytic turnover and contributes to sustained catalyst regeneration. This interpretation is also consistent with dynamic ligand‐exchange behavior previously proposed for analogous [Co(bpy)_3_]^2+^‐based photocatalytic systems [22, 23, 24]. In addition, balancing the amount of Co‐bpy relative to TiO_2_ is also important for the catalytic performance. As shown in Figure 1b, the CO production rate varied markedly with Co content (mol% relative to TiO_2_) with the bpy‐to‐Co fixed at 50:1, exhibiting a distinct volcano‐like trend. The optimal loading of 6 mol% Co^2+^ relative to TiO_2_ yielded the highest CO production rate of 16.52 μmol h^−1^, while both lower (Co 3 %: 10.94 μmol h^−1^) and higher (Co 10 %: 6.94 μmol h^−1^) loadings resulted in reduced activity.

Furthermore, in order to understand the role of Co‐bpy, which favored CO_2_RR compared to HER, the electrochemical experiments were conducted. As shown in Figure 1c,d, the cyclic voltammetry was measured with a 3 electrode configuration of a carbon paper as a working electrode, a platinum plate as a counter electrode, and a Ag/AgCl wire as a reference electrode in an acetonitrile–water solution electrolyte. Under Ar purging, regardless of the presence of Co‐bpy, no critical faradaic reaction was observed. However, under a CO_2_‐saturated condition, a distinct reduction peak at 0.2 V_NHE_ and at −0.4 V_NHE_ appeared with Co‐bpy. The former peak corresponds to the reduction of the Co^2+^ metal center to Co^+^, and the latter peak indicates the dissociation of a single bpy ligand [16]. These sequential processes represent the initial activation of the Co‐bpy, generating an open coordination site at the Co center for subsequent catalytic reactions. Beyond the bpy dissociation, the cathodic current increased, consistent with CO production. On the other hand, under Ar purging, current densities increased as negatively increasing applied potential from −1.0 V_NHE,_ which indicates favorable HER using Co‐bpy without CO_2_ present. From these CV studies, the role of Co‐bpy was understood with two criteria: (1) Co‐bpy initiates CO_2_RR at a less negative potential range than HER, indicating favored CO_2_RR compared to HER, and (2) the turnover frequency of CO_2_RR, referred to as current densities, was much faster than HER [15].

Furthermore, to examine the interactions between Co‐bpy and the TiO_2_ photocatalyst, multiple consecutive photocatalyses were performed with and without Co‐bpy (3 mol% Co^2+^ relative to TiO_2_, bpy:Co = 50:1), as shown in Figure 1e. After each cycle of photocatalysis, the dispersed TiO_2_ catalyst was filtered and reused for the next measurements. During the 1^st^ and 2^nd^ runs of photocatalysis with Co‐bpy, the CO production rate was almost identical, representing this hybrid photocatalyst was stable. However, during the 3^rd^ run of the photocatalyst without adding Co‐bpy, CO production was perfectly suppressed; however, by additionally adding Co‐bpy in solution during the 4^th^ run, this CO_2_RR activity was obviously recovered. It indicates that this hybrid photocatalyst is only activated with TiO_2_ and Co‐bpy dispersed in the reacting solution rather than immobilized on the TiO_2_ surface. The optimized hybrid photocatalyst showed long‐term stability for at least 7 h, as shown in Figure 1f. As conducting postreaction characterizations using X‐ray diffraction spectroscopy (XRD), field emission scanning electron microscopy (FE‐SEM), X‐ray photoelectron spectroscopy (XPS), and Fourier transform infrared spectroscopy (FT‐IR), as shown in Figures S1, S2, S6, and S7, respectively, no noticeable changes in the crystal structure, morphology, and chemical states were observed.

Based on these observations, the proposed working mechanism of the hybrid photocatalyst is illustrated in Figure 1g. Upon light irradiation, photoexcited electrons generated in the TiO_2_ conduction band are efficiently injected into the Co–bpy complex. This directional electron injection activates Co‐bpy for highly selective CO_2_‐to‐CO conversion, while the remaining holes are scavenged by TEOA.

### 2.2 Thermodynamics Reaction Pathway for CO_2_RR Compared to HER

**FIGURE 2:**
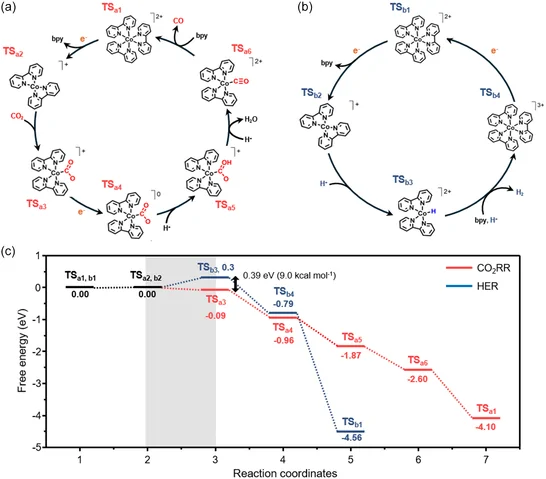
Proposed reaction mechanisms and thermodynamic profiles for a hybrid photocatalyst. Proposed catalytic cycles for (a) CO_2_RR and (b) HER. (c) Gibbs free energy diagram comparing CO_2_RR (red line) and HER (blue line) pathways.

To gain deeper theoretical insights into the reaction mechanisms and the origin of the selective promotion of CO_2_RR over HER, DFT analysis was performed for the proposed Co‐bpy intermediates after reductive activation. The proposed reaction pathways for CO_2_RR and HER are illustrated in Figure 2a,b, respectively. Because Co‐bpy complexes can access multiple spin states, spin state screening for doublet and quartet states was conducted for Co‐bpy, and the quartet‐derived pathway was selected as the representative thermodynamic pathway (Figure S8 and Table S1) [25]. Both CO_2_RR and HER are two‐electron processes driven by electrons transferred from TiO_2_; however, CO_2_RR involves a more complex six‐step route compared to the four‐step HER. While such complexity might typically make CO_2_RR less kinetically favored, the DFT energy profile (Figure 2c) of thermodynamic states (TSs) along reaction pathways reveals a clear thermodynamic preference.

During the transition from TS_a1, b1_ to TS_a2, b2_, photoexcited electrons from TiO_2_ are injected into Co‐bpy center with the simultaneous dissociation of a single bpy ligand [16]. Since both CO_2_RR and HER exhibit near‐zero Gibbs free‐energy changes (Δ*G*) for this step, this initial catalyst activation is thermodynamically facile and unbiased with no preference between the two reactions. However, as the reaction proceeds from TS_a2_ (or TS_b2_) to TS_a3_ (or TS_b3_) (gray region), the subsequent coordination of CO_2_ or H^+^ to the Co–bpy complex is energetically distinctive, where the H^+^ binding is uphill, while the CO_2_ binding is downhill. The calculated Δ*G* for CO_2_ binding is −0.09 eV, whereas the energy for H^+^ binding is higher at + 0.30 eV. In addition, the reaction energy barrier between TS_a2_ and TS_a3_ is obtained as 0.133 eV, which is small compared to the thermodynamic energy difference between TS_b2_ and TS_b3_ (Figure S9). Even though a more complete kinetic assessment would require consideration of the activation barriers for the relevant elementary reaction steps, the thermodynamic difference makes CO_2_ coordination more favorable, supporting the high CO_2_RR selectivity of the hybrid catalyst despite the inherent disadvantage of the complex multistep pathway [26].

Furthermore, following the CO_2_ binding, the transition from TS_a3_ to TS_a4_ in CO_2_RR involves the consumption of a second electron to convert [Co(bpy)_2_CO_2_]^+^ into [Co(bpy)_2_CO_2_]^0^. This reduction step thermodynamically stabilizes the CO_2_ adduct and lowers the overall energy of the system. Such stabilization drives the subsequent proton‐coupled reaction, ultimately releasing CO and completing the catalytic cycle. Altogether, these DFT calculations provide a comprehensive energetic landscape of the hybrid catalyst; they successfully demonstrate that the high selectivity for CO production is dictated by the thermodynamic advantage for CO_2_ coordination, while the subsequent electron transfers ensure the thermodynamic feasibility of the entire multistep reduction sequence.

### 2.3 Charge Dynamics of Photoexcited Electron Over a Hybrid Photocatalyst

To elucidate the mechanism behind the selective promotion of CO_2_RR in the hybrid photocatalyst, the charge carrier dynamics were analyzed using TAS, providing experimental access to the real‐time kinetic behavior of photoexcited charge carriers and transient photoinduced species. Building upon DFT insights that identify CO_2_ coordination as the energetically preferred pathway, TAS was employed to monitor the charge‐transfer dynamics and transient species evolution at the Co‐bpy center, complementing the correlation between thermodynamic preference and catalytic selectivity. Nanosecond TAS measurements were performed using a pump‐probe system equipped with an Nd:YAG laser (6–8 ns pulse width) as the excitation source and a 150 W Xenon lamp as the probe beam. Given that a sacrificial hole scavenger was used in each TA analysis to quench photogenerated holes, the detected signals were primarily attributed to the dynamics of photogenerated electrons.

**FIGURE 3:**
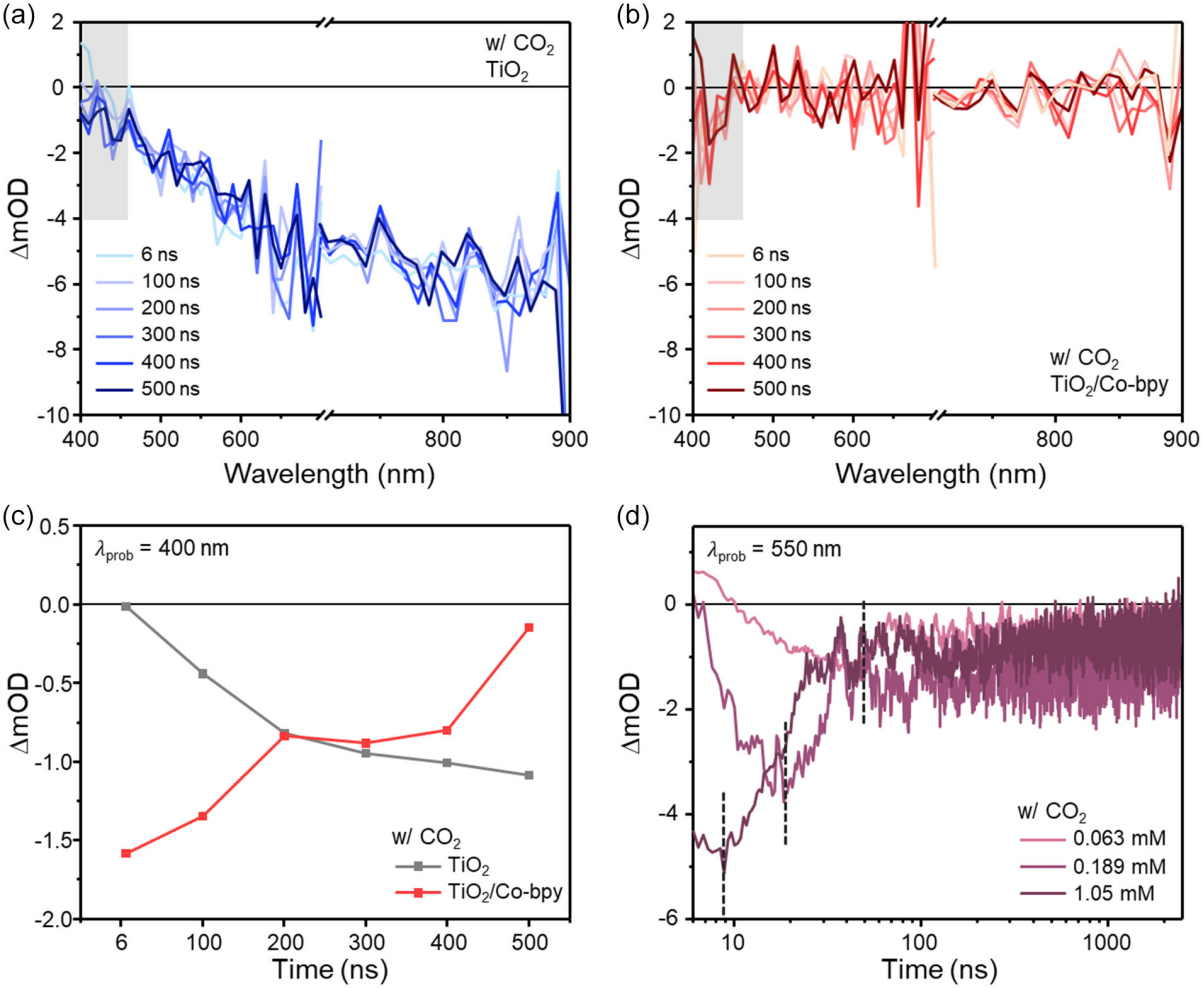
Transient absorption (TA) analysis of photoexcited charge carrier dynamics. TA spectra of (a) pristine TiO_2_ and (b) hybrid photocatalyst in the visible region (400–900 nm). (c) TA kinetic traces of pristine TiO_2_ and hybrid photocatalyst with 0.189 mM Co‐bpy monitored at 400 nm. (d) TA kinetics of TiO_2_/Co‐bpy hybrid photocatalyst at 550 nm with various concentrations of Co‐bpy (0.063, 0.189, and 1.05 mM; bpy:Co = 50:1), showing concentration‐dependent evolution of the transient feature. All measurements were performed under CO_2_ atmosphere.

In the pristine TiO_2_ system (Figure 3a), a broad ground‐state bleaching (GSB) signal rapidly appeared across the entire wavelength range at the initial stage (6 ns) and persisted beyond 500 ns, consistent with the typical behavior of long‐lived photoexcited electrons of TiO_2_ [27]. The presence of this broad GSB feature reflects the efficient generation and persistence of photogenerated electrons. In contrast, the hybrid photocatalyst (Figure 3b) exhibited an immediate quenching of this broad TiO_2_ electron signal, providing clear spectroscopic evidence of fast electron injection from TiO_2_ to the Co–bpy complex [28]. This rapid quenching indicates that Co‐bpy functions as an efficient electron acceptor, extracting photogenerated electrons from TiO_2_ on a nanosecond timescale.

Interestingly, a distinct transient absorption feature emerged around 400 nm (gray region) in the hybrid catalyst, which was absent in pristine TiO_2_ (Figure 3a,b). In particular, in the presence of Co‐bpy, a distinct transient feature appeared at around 400 nm, indicating the formation of a new photoinduced species. To further examine this difference, the temporal evolution of the signal at 400 nm was monitored from 6 ns to 500 ns ‍(Figure 3c). While the signal intensity for pristine TiO_2_ (gray line) increased over time due to continuous electron accumulation and overlapping contributions, the hybrid catalyst (red line) exhibited a distinct kinetic profile, characterized by a strong initial amplitude, followed by a gradual decay. This characteristic rise‐decay behavior indicates the formation of a transient species with distinct kinetics from the continuous charge accumulation observed in pristine TiO_2_. Importantly, this transient feature emerges only in the presence of Co‐bpy, indicating that its formation originates from interactions between TiO_2_ and the molecular catalyst. As corroborated by our DFT results, this new species is consistent with the formation of a reduced intermediate following electron injection from TiO_2_ to Co‐bpy and is closely associated with the CO_2_ reduction pathway [29]. Although the present TAS measurements do not allow unambiguous assignment of this transient feature to a specific molecular species, its exclusive appearance under a CO_2_ atmosphere supports its close association with the CO_2_ reduction pathway.

This finding is further supported by concentration‐dependent kinetic analysis probed at 550 nm (Figure 3d). Specifically, as the Co‐bpy concentration increases from 0.063 to 1.05 mM, the transient signal exhibits a progressively faster decay. The onset of this decay is highly sensitive to the catalyst loading: the signal begins to diminish at 50 ns for the 0.063 mM concentration, significantly accelerating to 18.4 ns at 0.189 mM and further to 8.8 ns at the highest concentration of 1.05 mM. This clear correlation between increasing Co‐bpy concentration and both the earlier onset and more rapid decay kinetics indicates that the transient feature is strongly influenced by catalyst loading. In addition, the absolute amplitude of the transient signal becomes larger with increasing concentration, indicating a greater contribution of the corresponding transient species. This simultaneous increase in amplitude and the shift toward an earlier decay onset are consistent with the formation of a Co‐bpy‐associated transient species under a CO_2_ atmosphere. The concentration‐dependent evolution of this feature further indicates that its dynamics are influenced by catalyst loading.

To further validate the reaction‐specific behavior of the hybrid photocatalyst, TAS analysis was performed to investigate the charge carrier dynamics under an N_2_ atmosphere (Figure S10), which revealed that only HER occurs, in contrast to the results presented in Figure 3 for the CO_2_ atmosphere. The spectral data in Figure S10 show a consistent trend with the CO_2_RR system in terms of initial charge separation; however, the distinct intermediate feature around 450 nm, which was prominent under CO_2_, was not observed under N_2_ conditions. This reaction‐dependent behavior was further elucidated by the kinetic profiles shown in Figure S11. As shown in Figure S11a, the bleaching signal of TiO_2_ is significantly suppressed upon the introduction of the Co‐bpy catalyst even under N_2_, indicating that the initial electron injection from TiO_2_ to the molecular catalyst remains highly efficient regardless of the reactant gas.

To distinguish the intrinsic effect of the reaction atmosphere from the catalytic role of Co‐bpy, we first examine the charge carrier dynamics of pristine TiO_2_ under N_2_ and CO_2_ conditions (Figure S11b and Table S2). In both cases, TiO_2_ exhibits long‐lived electron signals, indicating substantial charge accumulation within the semiconductor. Notably, under a CO_2_ atmosphere, the electron lifetime is moderately prolonged compared to N_2_, suggesting that CO_2_ can act as a weak electron acceptor, partially stabilizing photogenerated electrons and prolonging their lifetime. However, this behavior is fundamentally different from that observed in the hybrid system. In the presence of Co‐bpy, a significant kinetic divergence appears on the microsecond timescale. Under a N_2_ atmosphere, the decay kinetics are characterized by lifetime components of τ_1_ = 0.30 and τ_2_ = 4.34 μs, with the signal returning to the baseline within 10 μs, indicating that in the absence of CO_2_, the injected electrons decay rapidly following charge transfer. In contrast, the CO_2_ atmosphere significantly prolongs the charge carrier survival, as evidenced by the markedly increased lifetimes of *τ*_1_ = 1.18 and *τ*_2_ = 8.42 μs. The key distinction is that, while CO_2_ alone slightly prolongs electron lifetime in pristine TiO_2_ through slight suppression of recombination, it does not generate any new spectroscopic signature. In contrast, in the hybrid system, CO_2_ not only extends the carrier lifetime but also enables the formation of a distinct long‐lived intermediate, indicating the activation of a catalytic reaction pathway.

Comprehensively, the TAS analyses provide critical insights into the charge dynamics of the hybrid photocatalyst. Co‐bpy not only functions as an ultrafast electron acceptor to efficiently extract photogenerated electrons from TiO_2_ but also forms a CO_2_‐stabilized, long‐lived intermediate state required for the multistep CO_2_RR. The observed kinetics are consistent with the preceding DFT calculations, which theoretically predicted that the reduced Co‐bpy intermediate is thermodynamically stabilized upon CO_2_ coordination, unlike the unstable H^+^ adduct. This synergistic interaction—combining rapid interfacial electron transfer with the thermodynamic stabilization of the active intermediate—contributes to the enhanced selectivity for CO_2_‐to‐CO conversion relative to the competing HER. Overall, this mechanism fundamentally explains the enhanced CO_2_‐to‐CO conversion performance of the hybrid photocatalyst shown in Figure 1a.

### 2.4 Effect of Modifying the Electronic Structure of Molecular Catalyst

**FIGURE 4:**
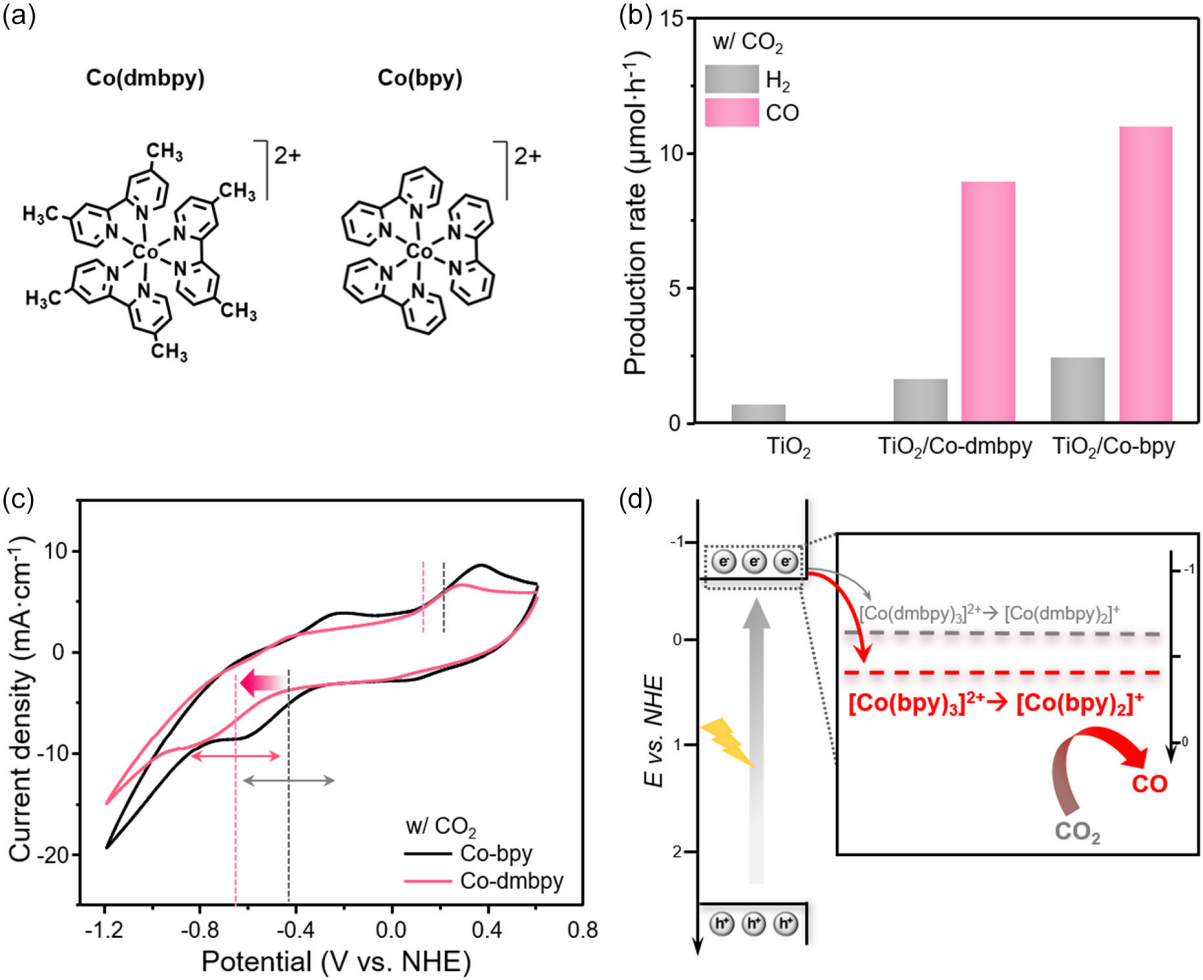
Effect of ligand electronic structure on photocatalytic and electrochemical properties. (a) Molecular structure of [Co(dmbpy)_3_]^2+^ (Co‐dmbpy) and [Co(bpy)_3_]^2+^(Co‐bpy). (b) Photocatalytic CO_2_RR performance for pristine TiO_2_ and hybrid catalysts incorporating Co‐dmbpy or Co‐bpy. (c) Cyclic voltammograms of Co‐bpy and Co‐dmbpy under CO_2_ saturation. (d) Energy level diagram illustrating the band alignment between TiO_2_ and the molecular complexes (Co‐bpy and Co‐dmbpy).

Having established that bpy dissociation is the prerequisite for generating an open Co coordination site and that CO_2_ coordination at this site is thermodynamically preferred over H^+^ adsorption, we next examined how modification of the ligand electronic structure influences activation and photocatalytic CO_2_RR activity. The effect of the ligand in the Co^2+^ complex within the hybrid photocatalyst was examined and is shown in Figure 4a. Here, two ligands, 4,4′‐dimethyl‐2,2′‐bipyridyl (dmbpy) and bpy, were used with Co^2^ to form the molecular catalysts [Co(dmbpy)_3_]^2+^ (Co‐dmbpy) and Co‐bpy, respectively. Then, photocatalytic CO_2_RR was conducted using TiO_2_ alone and hybrid photocatalysts comprising Co‐dmbpy and Co‐bpy, where the ratios of ligand‐to‐Co^2+^ and Co complex‐to‐TiO_2_ were set to be 50:1 and 3 mol%, respectively (Figure 4b). While both hybrid photocatalysts exhibited significantly higher CO production than pristine TiO_2_, the Co‐bpy hybrid demonstrated superior CO_2_RR efficiency over the Co‐dmbpy hybrid.

To understand the major contribution of the ligand in the metal complex, CVs were conducted with Co‐bpy or Co‐dmbpy under CO_2_ purging (Figure 4c). The two critical peaks representing the Co^2+^/Co^+^ reduction and ligand dissociation were negatively shifted with Co‐dmbpy compared to those with Co‐bpy. This shift is attributed to the methyl groups in dmbpy, which have a stronger electron‐donating ability than the terminal hydrogens in bpy [30, 31, 32]. This electron‐donating effect increases the electron density at the Co center, making it thermodynamically more difficult to accept an additional electron. Furthermore, it strengthens the Co–N bond, hindering the subsequent ligand dissociation. Because this dissociation is a prerequisite for creating an open active site for CO_2_, the hindered activation leads to a lower catalytic current density for Co‐dmbpy, which corresponds well to its lower CO production rate, as shown in Figure 4b.

Based on these electrochemical results, the band alignment of TiO_2_ with the metal complexes is illustrated in Figure 4d. Although the photoexcited electrons in TiO_2_ have sufficient thermodynamic potential to reduce CO_2_, the bare reaction is kinetically sluggish due to rapid charge recombination. The metal complex addresses this by serving as an electron‐transfer bridge, as its ligand dissociation energy level is favorably located between the conduction band minimum (CBM) of TiO_2_ and the theoretical CO_2_/CO redox potential. Notably, due to the aforementioned negative potential shift, the corresponding energy level of Co‐dmbpy is positioned higher (more negative) than that of Co‐bpy. Considering this band diagram, Co‐bpy maintains a more optimal energy gap with the TiO_2_ CBM, providing a more energetically favored driving force for photoexcited electron injection compared to Co‐dmbpy [33, 34, 35].

To further verify our ligand‐tuning approach, we examined several other candidates. First, 4,4′‐dimethoxy‐2,2′‐bipyridyl (dmobpy) was tested; however, the Co‐dmobpy hybrid exhibited negligible photocatalytic activity. This is directly attributed to an improper energy level alignment with the semiconductor, lacking a sufficient thermodynamic driving force for interfacial electron injection [36]. On the other hand, candidates such as 4,4′‐dinonyl‐2,2′‐bipyridyl (dnbpy) and 4,4′‐dibromo‐2,2′‐bipyridyl (dbbpy) possessed theoretically ideal energy levels but failed due to poor stability, quickly degrading under continuous illumination [37]. These results clearly demonstrate that a high‐performance hybrid photocatalyst requires both appropriate thermodynamic band alignment and robust photochemical stability. Moving forward, optimizing the interplay between the core metal, ligand, and semiconductor will be the key to designing durable and efficient artificial photosynthetic systems [38].

## 3 Conclusion

In summary, we demonstrated a highly efficient and selective TiO_2_/[Co(bpy)_3_]^2+^ hybrid photocatalyst for CO_2_ reduction. The optimized catalyst achieved a substantial CO production rate of 16.52 μmol h^−1^, whereas bare TiO_2_ predominantly produced H_2_ with no CO formation. Using this well‐defined hybrid system, we present converging mechanistic evidence from electrochemical analysis, DFT, and TAS, each addressing a distinct and consecutive elementary step of the photocatalytic cycle.

DFT calculations support the conclusion that CO_2_ coordination at the cobalt center is thermodynamically preferred over proton adsorption (Δ*G* = −0.09 eV vs. +0.30 eV), consistent with the high CO selectivity of the hybrid photocatalyst. Furthermore, TAS provides strong support for fast electron injection from TiO_2_ to Co‐bpy and reveals a CO_2_‐dependent long‐lived transient species that is consistent with the formation of reduced intermediates during CO_2_ reduction. In addition, ligand modulation is consistent with the proposed coupling between the semiconductor and molecular catalyst, highlighting the importance of energy‐level alignment for catalytic performance. The convergence of these three independent lines of evidence—active site generation (CV), CO_2_ coordination selectivity (DFT), and charge‐transfer dynamics (TAS)—provides the basis for the proposed mechanistic framework, in which selective CO_2_ reduction is facilitated by the synergy between rapid interfacial electron transfer and stabilization of CO_2_‐specific transient intermediates.

These findings offer a useful framework for understanding photocatalytic selectivity and guiding the development of next‐generation solar‐to‐chemical conversion systems.

## 4 Experimental Section/Methods

### 4.1 Materials

P25 TiO_2_, cobalt chloride (CoCl_2_, 99.99%), and 2,2′‐bipyridine (C_10_H_8_N_2_, ≥99%) were purchased from Sigma–Aldrich. Tetrabutylammonium hexafluorophosphate (C_16_H_36_F_6_NP, >98%) and 4,4′‐dimethyl‐2,2′‐bipyridine (C_12_H_12_N_2_, >98%) were purchased from Tokyo Chemical Industry (TCI) Co., Ltd. Acetonitrile (CH_3_CN, 99.8%) was purchased from Daejung Chemicals, and TEOA (C_6_H_15_NO_3_, 99%) was purchased from Samchun Chemicals.

### 4.2 Characterization

The crystal structures of the samples were verified by XRD (Miniflex 600, Rigaku, Cu‐Kα radiation, *λ * = 0.154 nm) measurements. The data was collected in the scanning scope (2*θ*) from 5° to 90° at a stepwise 0.05°. Morphologies of the samples were investigated using field emission scanning electron microscopy (FE‐SEM; Verios G4UC, acceleration voltage of 15 keV, and Clean‐Energy Research Institute Cryo FIB‐SEM (NFEC‐2025‐03−304 629)). The light absorbance and bandgap energy of the samples were measured using UV–vis spectroscopy (UV‐2600i, Shimadzu). The chemical composition and oxidation state of the samples were examined using XPS (K‐Alpha+, Thermo Fisher, Al‐Kα radiation, *hν* = 486.6 eV, and Clean‐Energy Research Institute, NAP‐XPS (NFEC‐2025‐05−306 118)). The carbon 1s peak at 284.8 eV was used as the reference point for calibrating the binding energies. The molecular vibrations of the samples were characterized using FT‐IR (Invenio, Bruker).

### 4.3 Photocatalytic CO_2_ Reduction Reaction (CO_2_RR)

All chemicals were used as received without any additional purification. The photocatalytic CO_2_RR were performed in a quartz reactor under light irradiation from a 400 W Hg lamp (Spectra Physics, 500 HX) without a cut‐off filter. For each sample, 20 mg of the photocatalyst (P25 TiO_2_) was dispersed in 21 mL of mixed solution containing acetonitrile (ACN) as a solvent, TEOA as a sacrificial agent, and deionized (DI) water as a proton source (4:1:1; v/v/v), with the addition of CoCl_2_ (3, 6, and 10 mol% of Co^2+^ relative to TiO_2_) and 2,2′‐bipyridine (bpy) or 4,4′‐dimethyl‐2,2′‐bipyridine (dmbpy) (ligand:Co = 50:1). Before every reaction, the slurry was thoroughly mixed via ultrasonication for 10 min and then purged with high‐purity CO_2_ (99.999%) for at least 30 min under magnetic stirring.

For the formation of Co‐bpy, specific amounts of cobalt precursor and bpy were simply added to the reaction solution, enabling the self‐assembly of the Co–bpy complex, as verified by the UV–vis absorbance data.

Control experiments were conducted with and without light, CO_2_, and TEOA using TiO_2_/Co‐bpy. Additional tests with TiO_2_ alone and Co‐bpy alone were conducted under standard photocatalytic CO_2_RR conditions with light, CO_2_, and TEOA.

To establish that the produced CO originates from the CO_2_, a ^13^CO_2_ isotope‐labeling experiment was conducted using DEMS (HPR‐40 Hiden Analytical, UK). An electron energy of 70 eV was used for the ionization of all species with an emission current of 50 μA. The photocatalytic reaction solution was placed in a PEEK cell, and the gas outlet of the cell was connected to the DEMS system through a sealed line. The solution was first purged with Ar for 30 min to remove the dissolved gases and residual gaseous impurities. After establishing a stable background signal, ^13^CO_2_ was introduced into the cell, followed by light irradiation to initiate photocatalytic CO_2_ reduction. DEMS continuously monitored the following gaseous products generated during the reaction: *m*/*z* = 2 (H_2_), 28 (^12^CO), 29 (^13^CO), 44 (^12^CO_2_), and 45 (^13^CO_2_).

### 4.4 Quantification of Products

The accumulated gaseous products in the headspace were analyzed using a gas chromatography (GC; ChroZen, Youngin Chromass) system. A sample volume of 0.2 mL was collected and injected manually into the GC using a syringe. The system included a Porapak N column (1/8", 10 ft, 80/100 mesh) and a Molecular Sieve 13X column (1/8", 3 ft, 45/60 mesh) for detecting H_2_, CH_4_, CO, CO_2_, C_2_H_6_, C_2_H_4,_ and C_2_H_2_. Detection was carried out with a thermal conductivity detector (TCD), a flame ionization detector (FID), and a methanizer. Argon served as the carrier gas at a flow rate of 20 mL min^−1^. The GC was routinely calibrated using standard gas mixtures under standard conditions (1 atm, 298 K).

### 4.5 Electrochemical Measurements

All electrochemical measurements were conducted in a one‐compartment cell with a three‐electrode system, controlled using a potentiostat (MP2F, Zive). A carbon paper (1 × 1 cm^2^ of geometric area), a platinum plate, and an Ag/AgCl wire electrode were utilized as the working electrode, the counter electrode, and the reference electrode, respectively. The electrolyte contains 3 mM of CoCl_2_ and 4 equivalents of bpy in a 0.1 M tetrabutylammonium hexafluorophosphate (TBAPF_6_)/ACN and DI water (9:1; v/v) solution. Before every measurement, Ar or CO_2_ gas was purged for at least 30 min. All potentials were converted to the normal hydrogen electrode (NHE) using the equation below.

E(vs.NHE)=E(vs.Ag/AgCl)+0.21

Cyclic voltammograms (CVs) were recorded while varying the potential from 0.8 to −1.3 V_NHE_ at scan rates of 150 mV s^−1^.

### 4.6 Nanosecond TAS

Nanosecond TAS data were obtained through a pump and probe ns‐transient system. This system includes a transient absorption spectrometer and a regenerative amplified Nd:YAG laser (EL‐YAG) with a pulse width of 6–8 ns, capable of generating both visible (532 nm) and UV pulses (355 nm) through a third harmonic generator. The probe beam is generated from a 150 W Xenon lamp, which reflects off the powdered sample, passes through a monochromator, and then reaches a PMT‐980 photodiode detector. To simultaneously collect data on two different time scales, we utilize the comprehensive L900 spectrometer software (V9.4.6) package, and the ns‐s signal is sampled using an oscilloscope (Tektronix MDO3022, Beaverton, OR, USA). Excitation fluences were measured using a pyroelectric energy sensor.

### 4.7 Computational Analysis

We optimized molecular structures employing DFT calculations at the B3LYP/def2‐SVP level with a CPCM solvent model including Grimme’s DFT‐D4 correction using ORCA [39]. Frequency calculations for Gibbs free energies were performed at the B3LYP/def2‐TZVP level. The CHE framework enabled systematic computation of Gibbs free energies, integrating electronic, zero‐point, entropy, and applied potential effects. The Gibbs free energy (Δ*G*) was determined using the following thermodynamic formulation:

$$\text{Δ}G=\text{Δ}E_{\mathrm{ele}}+\text{Δ}ZPE-T\text{Δ}S+\text{Δ}G_{U}$$

Herein, Δ*E*_ele_ indicates the electronic energy obtained from DFT calculations. Zero‐point energy (ΔZPE) and entropic contributions (*T*Δ*S*), derived from vibrational analysis, serve as thermal correction parameters at room temperature. $\text{Δ}G_{U}=-\mathrm{ne}U$ incorporates the absorbed photon energy for the excitation of the semiconductor (TiO_2_) and the applied electrode potential with respect to CHE (*U* = −2.28 V), where n is the number of electrons. From the fundamental condition of the CHE model $\left(H^{+}+e^{-}\to \frac{1}{2}H_{2}\right)$, we obtained the Gibbs free energy of an electron, G(e^−^), as $-\mathrm{eU}=G\left(H^{+}\right)+G\left(e^{-}\right)-\frac{1}{2}G\left(H_{2}\right)$ given *U*. Consistent with established literature, the free energy of a proton, G(H^+^), was assigned a value of –265.9kcal/mol [40].

## Funding

This study was supported by the National Research Foundation of Korea (RS‐2025‐25400520 and RS‐2023‐NR119931) and the Korea Institute of Energy Technology Evaluation and Planning (RS‐2025‐02654073).

## Conflicts of Interest

The authors declare no conflicts of interest.

## Supporting information

Supplementary Material

## Data Availability

The data that support the findings of this study are available from the corresponding author upon reasonable request.
