# Supplementary material for: Unveiling the Mechanisms of Photocatalytic CO2 Reduction Reaction in Semiconductor/Metal Complex Hybrid Photocatalyst
Source: Small Sci. 2026 Sep 18;6(9):e70407. doi: 10.1002/smsc.70407 (PMC13588002; doi:10.1002/smsc.70407)
Supplement: Supplementary file 1 [file SMSC-6-e70407-s001.pdf]

Supporting Information

Unveiling the Mechanisms of Photocatalytic CO_2 Reduction Reaction in Semiconductor/Metal Complex Hybrid Photocatalyst

Dasom Kim, Gayoung Ham, Jonghwan Lee, Yeseul Jo, Jeongu You, Seung Kyu Min, Hyojung Cha*, Youn Jeong Jang**

D. Kim, Y. Jo, J. You, Y. J. Jang
Department of Chemical Engineering, Hanyang University, Seoul, 04763, Republic of Korea
E-mail: yjang53@hanyang.ac.kr

G. Ham, H. Cha
School of Energy Engineering, Kyungpook National University, Daehak-ro 80, Buk-gu, Daegu, 41566, Republic of Korea
E-mail: hcha@knu.ac.kr

J. Lee, S. K. Min
Department of Chemistry, Ulsan National Institute of Science and Technology, Ulsan, 44919, Republic of Korea
E-mail: skmin@unist.ac.kr

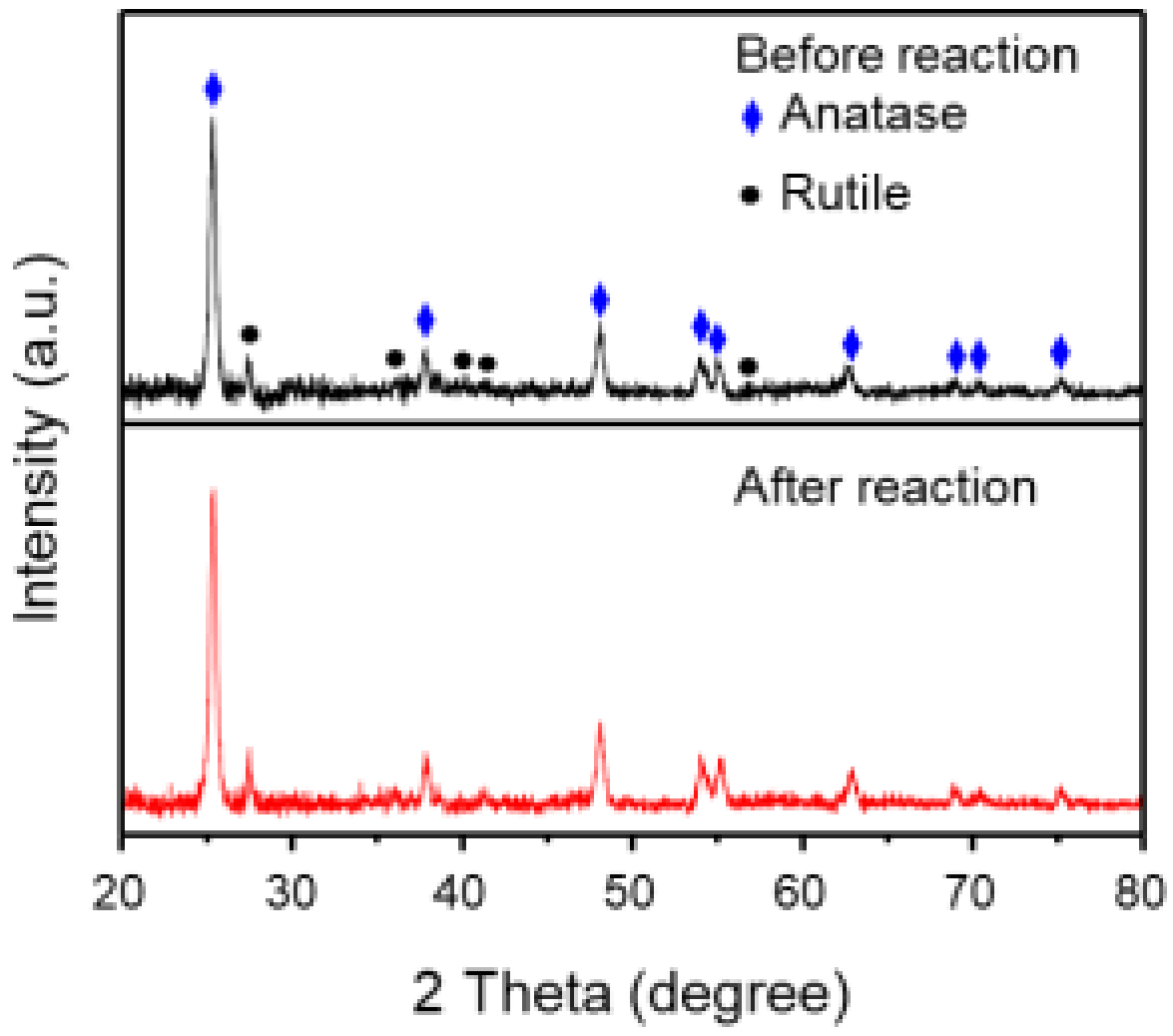

Figure S1. XRD patterns of TiO_2 before and after the photocatalytic reaction.

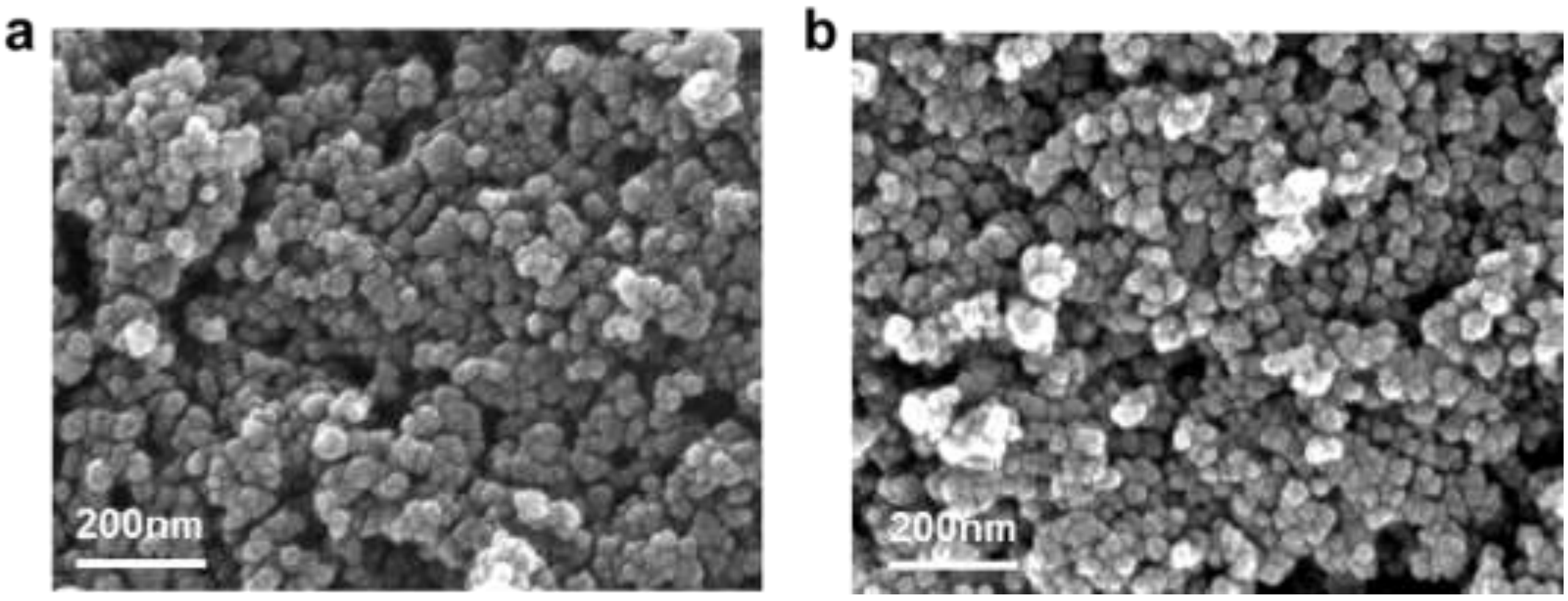

Figure S2. FE-SEM images of TiO_2 (a) before and (b) after the photocatalytic reaction.

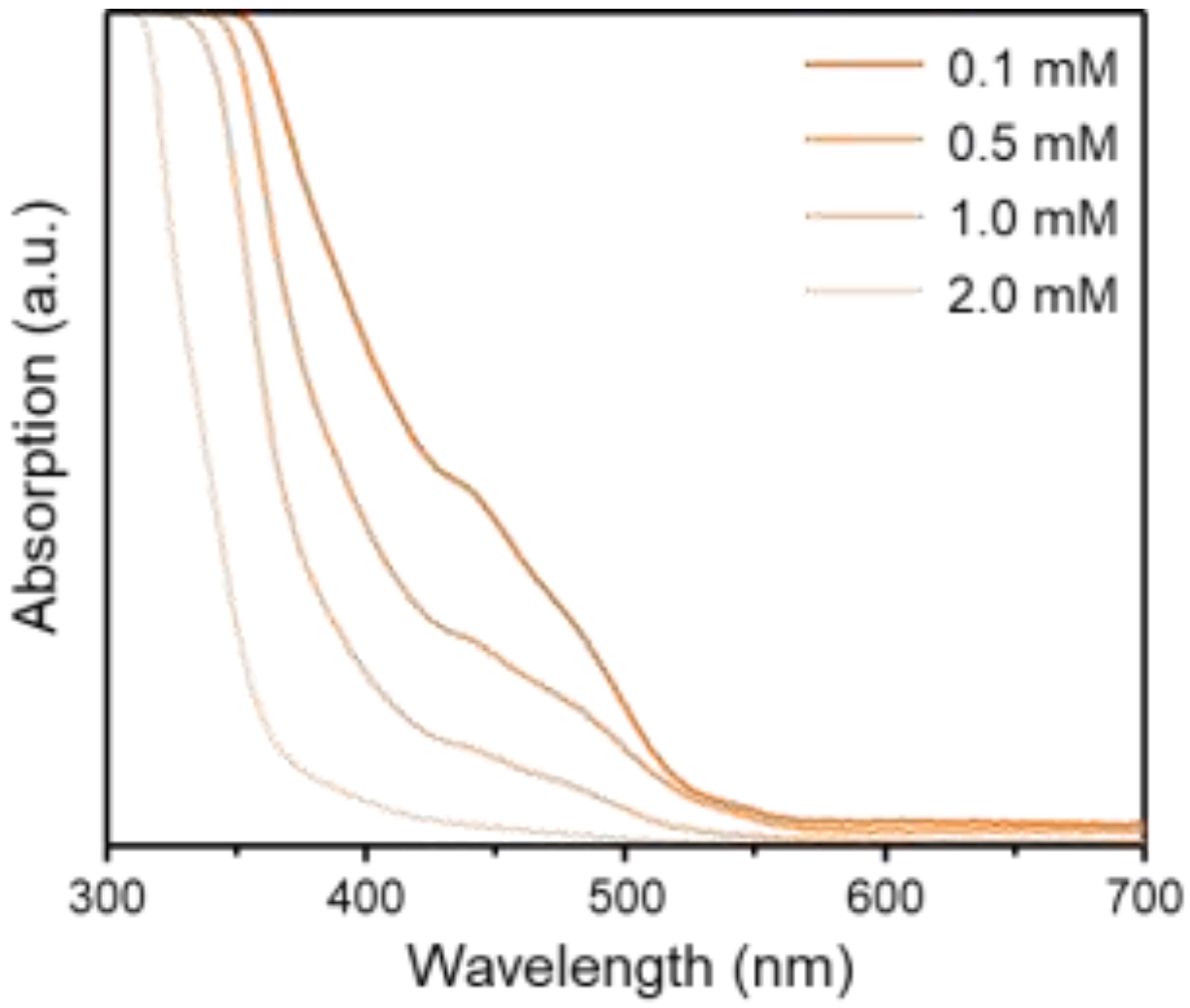

Figure S3. UV-Vis diffuse absorption spectra of self-assembled Co-bpy. Absorption spectra recorded at various Co^{2+} concentrations (0.1-2.0 mM; bpy:Co=50:1) in an ACN/H_2O mixture, confirming the successful formation of the molecular catalyst.

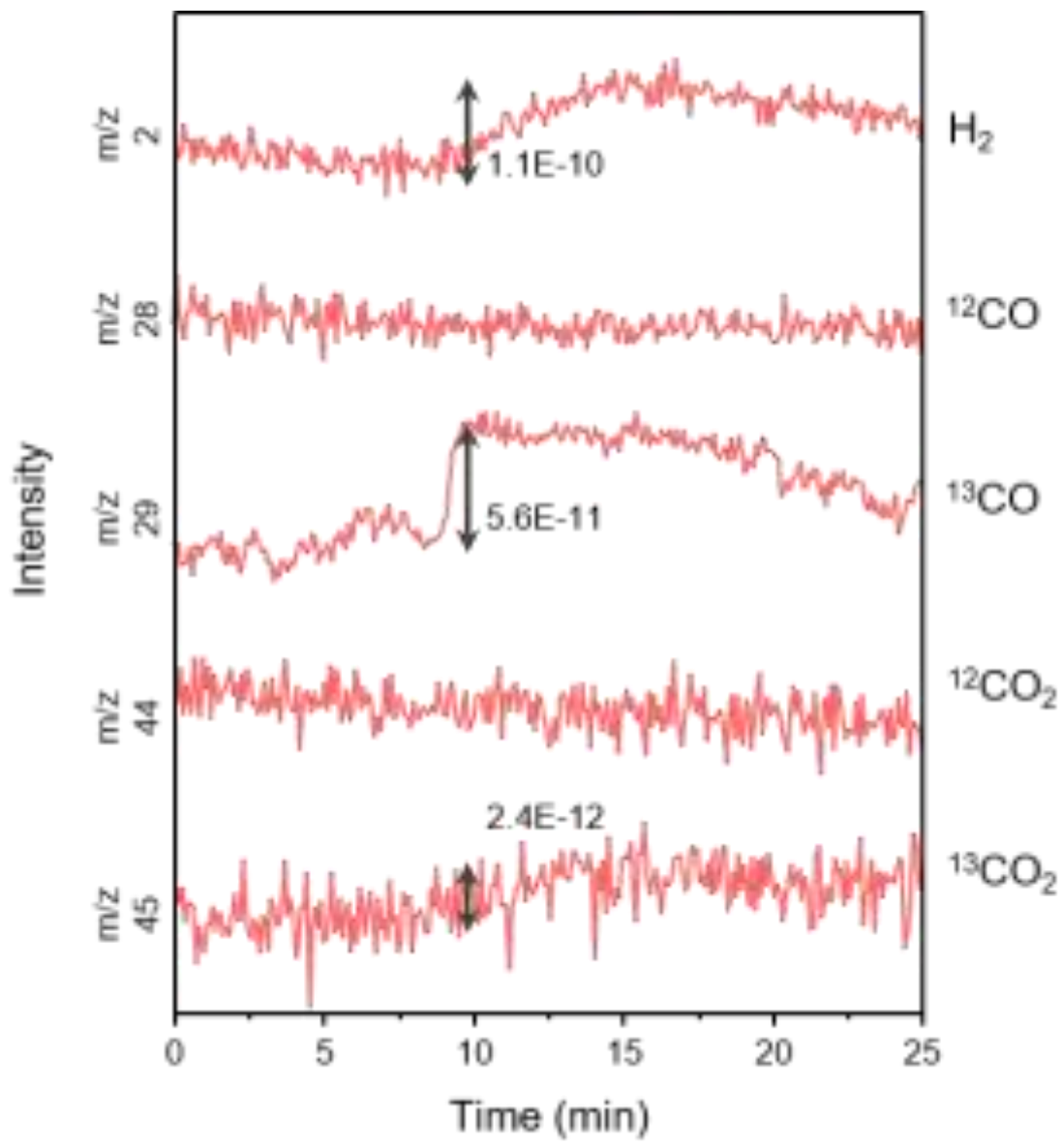

Figure S4. DEMS signals during the $^{13}CO_2$ isotope-labeling experiment for photocatalytic CO_2 reduction using hybrid photocatalysts.

The isotope-labeling experiment was conducted under the optimized reaction conditions, using Co^{2+} at 6 mol% relative to TiO_2 and a bpy/Co^{2+} molar ratio of 50:1. Under $^{13}CO_2$, signals for $^{13}CO_2$ and ^{13}CO were detected, whereas no $^{12}CO_2$ or ^{12}CO signals were observed. This confirms that the produced CO originated from the supplied CO_2 and excludes a detectable contribution from other carbon-containing components or contaminants

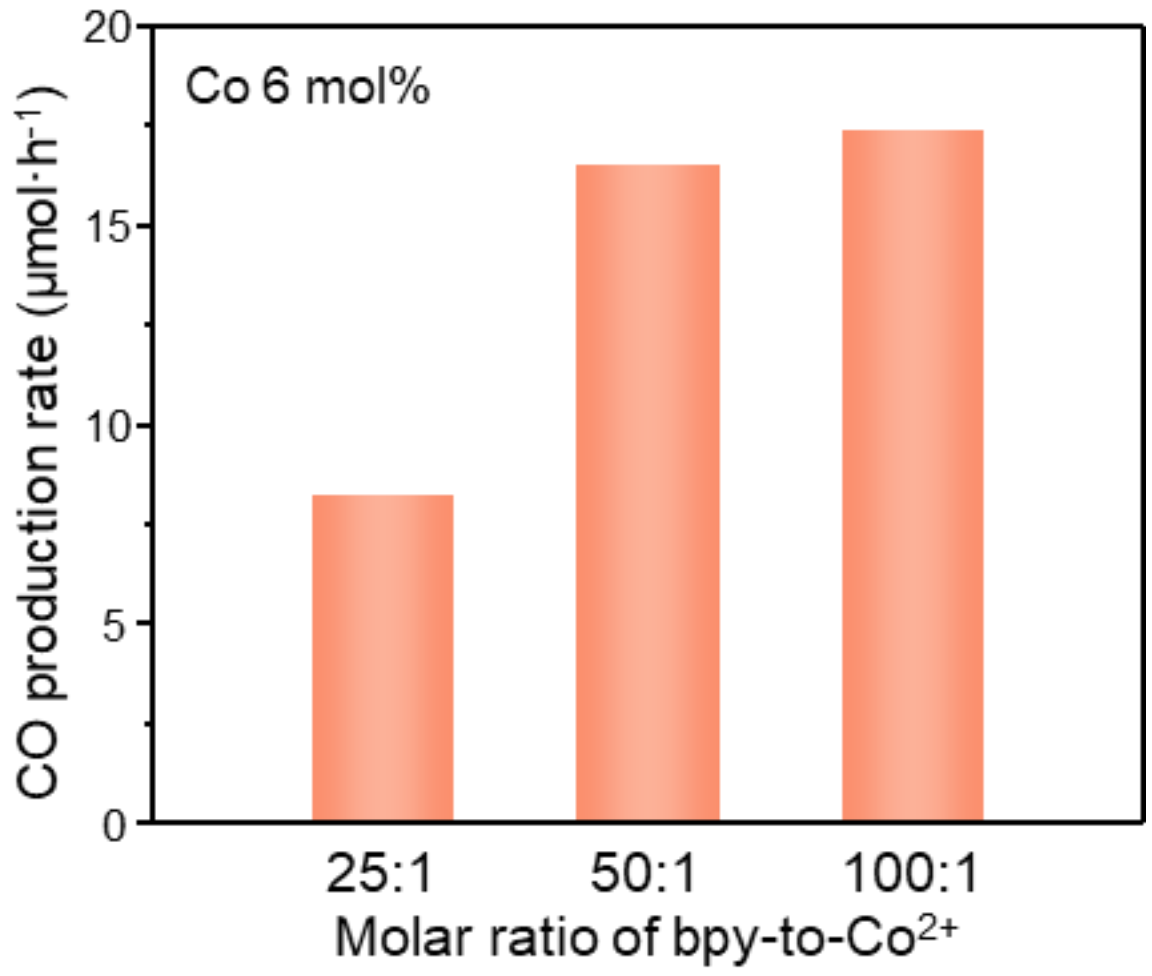

Figure S5. CO production rates as a function of bpy-to-Co^{2+} ratio at a fixed Co^{2+} content of 6 mol% relative to TiO_2.

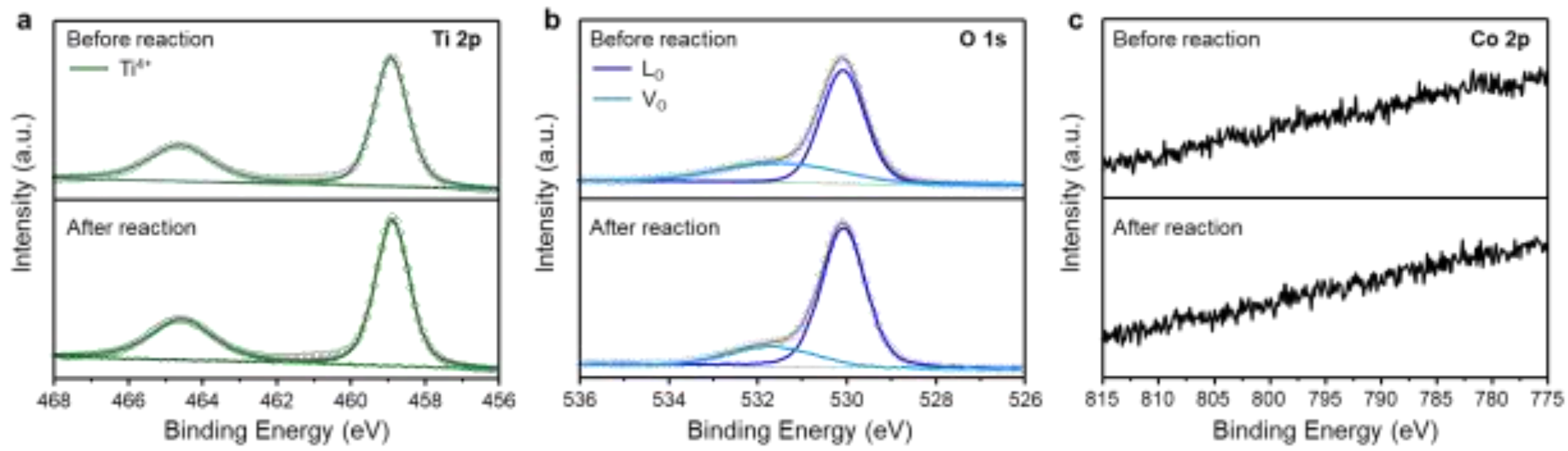

Figure S6. XPS spectra of (a) Ti 2p, (b) O 1s, and (c) Co 2p of TiO_2 before (top) and after (bottom) the photocatalytic reaction.

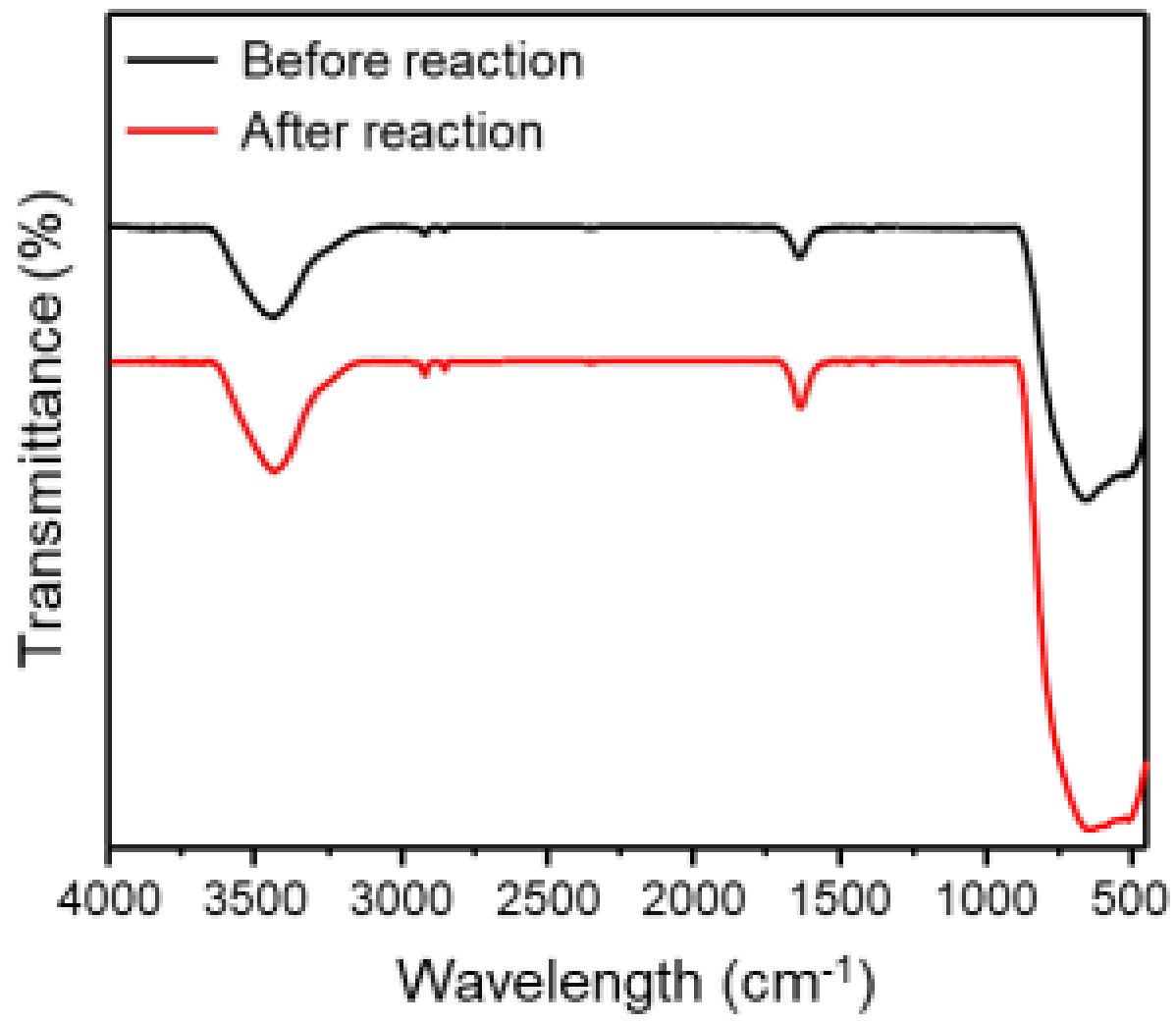

Figure S7. FT-IR spectra of TiO_2 before and after the photocatalytic reaction.

Spin state screening of Co-bpy intermediates along the CO_2RR and HER pathways

Table S1. Spin multiplicities considered for the optimized thermodynamic intermediates along the proposed CO_2RR and HER pathways of the Co-bpy catalyst. Indices 1–3 correspond to the CO_2RR pathways, whereas indices 4–6 correspond to the HER pathways. Reaction coordinates 1–6 denote the sequential intermediates along each pathway, and 1′ denotes the regenerated catalyst state.

Reaction	Index	1	2	3	4	5	6	1'
CO_2RR	1	Doublet	Singlet	Singlet	Doublet	Doublet	Doublet	Doublet
	2	Doublet	Triplet	Triplet	Doublet	Doublet	Doublet	Doublet
	3	Quartet	Triplet	Triplet	Quartet	Quartet	Quartet	Quartet
HER	4	Doublet	Singlet	Singlet	Singlet	-	-	Doublet
	5	Doublet	Triplet	Triplet	Triplet	-	-	Doublet
	6	Quartet	Triplet	Triplet	Triplet	-	-	Quartet

We performed spin state screening for the Co-bpy intermediates because Co-bpy complexes can access multiple spin states. Both doublet- and quartet-based initial states were considered, and the possible CO_2RR and HER pathways were evaluated for each spin manifold. The optimized doublet- and quartet-derived pathways showed very similar ΔG values for the thermodynamically demanding CO_2 binding (pathways 2 and 3) and H^+ binding steps (pathways 5 and 6). This indicates that the qualitative thermodynamic preference for CO_2 binding over proton binding is not significantly affected by the initial spin state assignment **(Figure S8)**. However, in this model, the quartet initial state is lower in Gibbs free energy than the doublet initial state by 2.786 kcal/mol, corresponding to a dominant quartet population (99.1%) at room temperature based on a Boltzmann distribution. Therefore, the main thermodynamic discussion focuses on the quartet pathway.

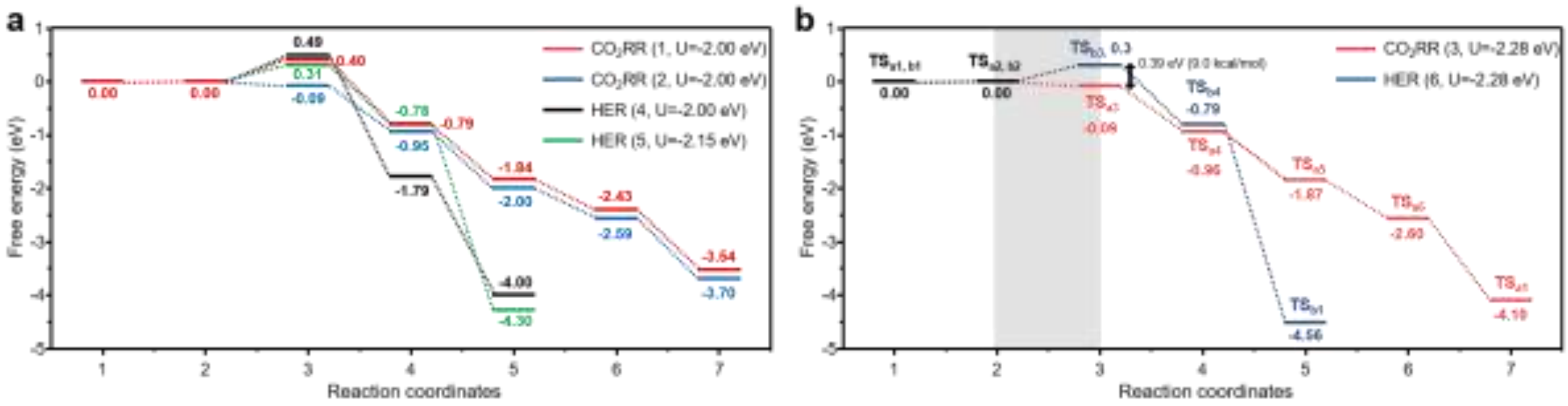

Figure S8. Spin state-dependent Gibbs free energy profiles for the proposed CO_2RR and HER pathways of the Co-bpy catalyst. (a) Doublet-derived pathways and (b) quartet-derived pathways.

Potential energy scan analysis of CO_2 binding

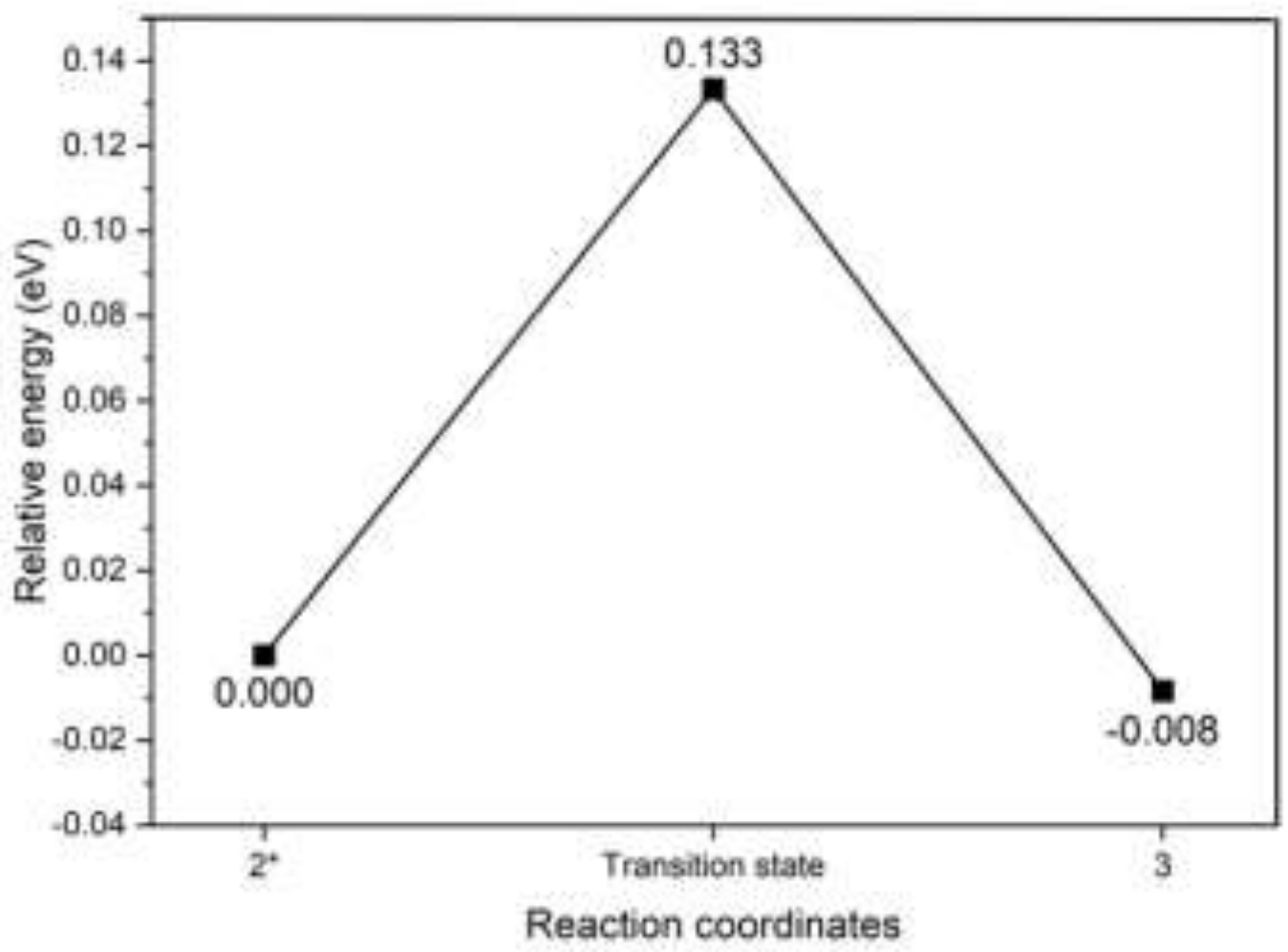

Figure S9. Relative energy profile for CO_2RR pathway from TS_{a2} to TS_{a3}. The reaction energy barrier is 0.133 eV (3.07 kcal/mol).

We examined whether a meaningful energy barrier between TS_{a2} and TS_{a3} could be identified for the thermodynamically demanding CO_2 binding. To this end, constrained geometry optimizations were performed first to determine the initial structure for transition state calculations by varying the distance between CO_2 and the Co center. For the CO_2RR pathway, $[Co(bpy)_2]^+$ and CO_2 becomes a pre-complex (2*) and then, the final $[Co(bpy)_2CO_2]^+$ complex (3) is produced via the transition state with a barrier of 0.133 eV (3.07 kcal/mol) which is smaller than the energy difference between thermodynamic states (TS_{b2}/TS_{b3}) of HER pathway.

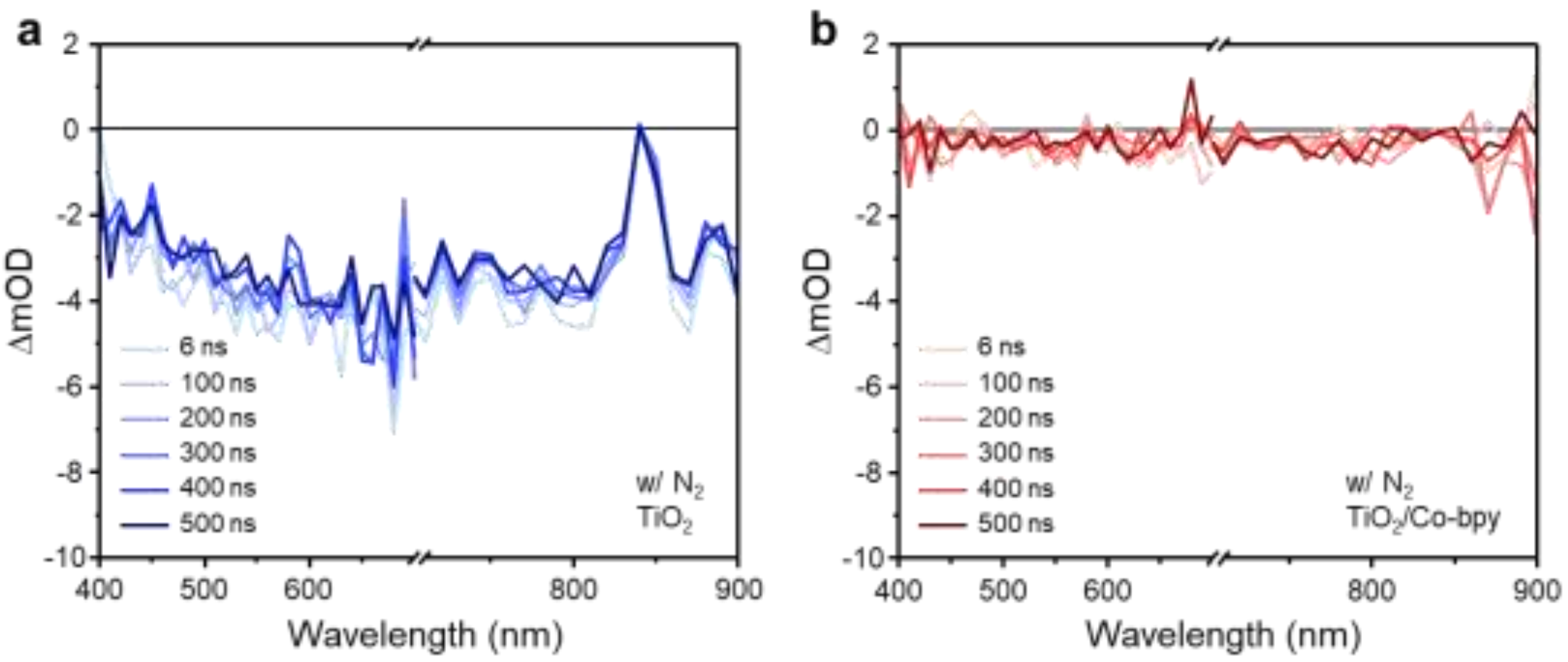

Figure S10. Transient absorption (TA) spectra of (a) pristine TiO_2 and (b) hybrid photocatalyst in the visible region (400-900 nm). All measurements were performed under N_2 atmosphere.

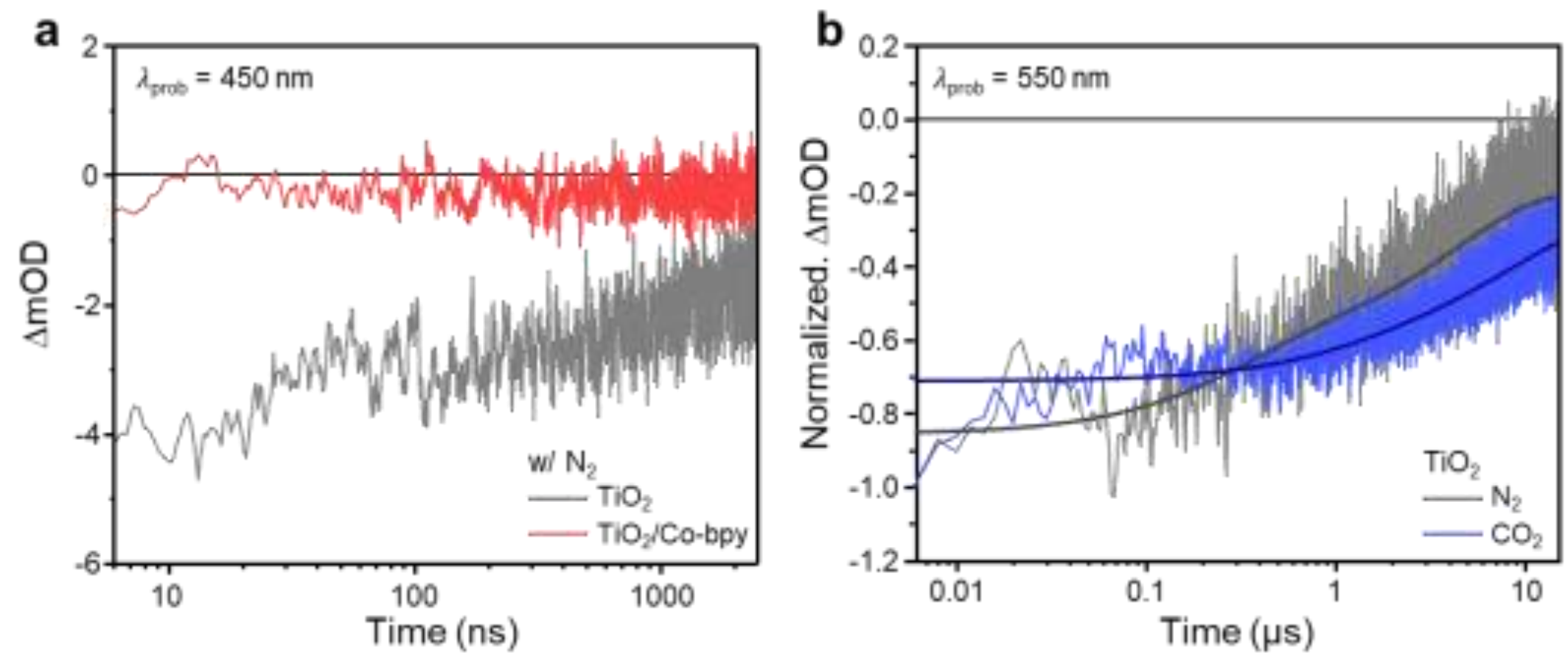

Figure S11. (a) TA kinetics of TiO_2 and the TiO_2/Co-bpy measured under N_2 atmosphere at 450 nm. (b) Normalized TA kinetics of pristine TiO_2 under N_2 and CO_2 atmosphere at 550 nm. Solid lines represent the bi-exponential fitting results.

Table S2. Kinetics parameters of TA decay for pristine TiO_2 under N_2 and CO_2 atmospheres. The lifetime fitting the kinetics at 550 nm with a bi-exponential decay function: $y = A_1 \times \exp(-t/t_1) + A_2 \times \exp(-t/t_2)$.

**Atmosphere**	**A_1**	**τ_1 (μs)**	**A_2**	**τ_2 (μs)**
N_2	-0.23	0.30	-0.42	4.34
CO_2	-0.08	1.18	-0.36	8.42

Equilibrium geometry and corresponding free energy

H_2

Gibbs free energy = -1.17528595 Hartree

H	0.00000000000000	0.00000000000000	0.38080091416901
H	0.00000000000000	0.00000000000000	-0.38080091416901

==

H_2O

Total electronic energy = -76.43365605 Hartree

O	0.00000000000000	-0.00000000378775	0.12454021348198
H	0.00000000000000	0.75499161618379	-0.48291410827230
H	0.00000000000000	-0.75499161239604	-0.48291410520968

==

CO

Gibbs free energy = -113.32590230 Hartree

C	0.00000000000000	0.00000000000000	-0.64641499866907
O	0.00000000000000	0.00000000000000	0.48386899866907

==

CO_2

Gibbs free energy = -188.60107784 Hartree

C	-0.00000001845360	-0.00000000738611	0.00000001824978
O	0.00000000922680	0.00000000369305	1.16317973654629
O	0.00000000922680	0.00000000369305	-1.16317975479606

==

bpy

Gibbs free energy = -495.18411494 Hartree

C	-1.47415216790240	1.18106505126167	-0.27361591060947
C	-3.49864100230383	-0.08093371485545	0.01287616760494
C	-2.69217286712373	-1.19583675038341	0.26540887545998
N	-1.35873190568434	-1.15946752608340	0.25797323272065
C	-0.74723974702081	0.00835075670371	-0.00221236875614
C	1.47414033217410	1.18107805478451	0.27362828617871
C	0.74723648555409	0.00835768925332	0.00222890110967
N	1.35873922873708	-1.15945868391831	-0.25794052654727
C	2.69218044621241	-1.19581981804536	-0.26536414096839
C	3.49863970512954	-0.08091060364502	-0.01283172393668
H	-0.95972419102543	2.11399789922357	-0.50780461742264
H	-4.58718911771158	-0.16889756675735	0.03149485093533
H	-3.15464878769867	-2.16570862185292	0.48639981883274
H	0.95970647763831	2.11401054031480	0.50780504273517
H	3.15466551095834	-2.16569088050137	-0.48634048597923
H	4.58718818524122	-0.16886920500820	-0.03143799823599
C	2.86821365712719	1.13293902157115	0.26519766315944
C	-2.86822514094487	1.13291733392718	-0.26517220845440
H	3.45240596485037	2.03142921404965	0.47985138185259
H	-3.45242506620699	2.03140180996123	-0.47982923967902

===

TS_{a1} (Doublet)

Gibbs free energy = -2868.12008871 Hartree

Co	0.01257651892820	0.03916021974557	0.00457445269351
C	3.20036413967296	2.99307176642329	-1.93810594174247
C	3.56939517569320	2.05886768654618	-0.97193597063876
C	2.60193119599975	1.19787355602460	-0.44174048027074
N	1.31533608766291	1.25913969663565	-0.86097910306974
C	0.95889356765779	2.16010063071695	-1.78688717345709
C	1.86965910653178	3.04762584576788	-2.35422675488486
C	4.20549942967468	-0.14341979015161	0.99863988615047
C	4.38018625972961	-1.11553729290195	1.98400846700612
C	3.25809558985269	-1.73349728100475	2.53871082940918
N	1.83053656307650	-0.42256059556570	1.13572161234119
C	2.90486515643433	0.18162544602494	0.59411093831415

C	-4.33164772115318	1.30441797387073	-1.95181012992368
C	-3.76303084585803	1.93453534168315	-0.84447578133573
C	-2.46709070271457	1.57448323484594	-0.45531006093261
N	-1.76825141781257	0.63708627868670	-1.12277413360902
C	-2.31158892676464	0.03384923533231	-2.18203764190896
C	-3.59537229870445	0.33713178028088	-2.63827511454663
C	-2.24365943155420	3.30937571320700	1.37548270397422
C	-1.52458547480139	3.81833765604707	2.45553974826854
C	-0.34134090869506	3.18588473414342	2.83932718159836
C	0.07673152264436	2.06657400351525	2.12416183601229
N	-0.61694680936238	1.57551823730056	1.08783998589281
C	-1.76667332796557	2.17890857484901	0.70284195577831
C	1.99670345030038	-1.35216907193509	2.07862991091435
C	-1.05476586630533	-2.53613019657692	0.39086044634325
N	-1.10241531904668	-1.29531189313857	0.93486043648730
C	-1.86252269039935	-1.08156284638631	2.01776165599408
C	-2.61740044223850	-2.09172822207207	2.61063067277952
C	0.01655345853349	-3.84221620889150	-1.49892210766859
C	0.85969824918007	-3.83747081565714	-2.60912135516228
C	1.48034881561138	-2.64456756002589	-2.98057377158876
C	1.23790931096483	-1.49909412426208	-2.22493895013272
N	0.43160098213163	-1.50275558091088	-1.15461640185217
C	-0.18171362843284	-2.65488055449852	-0.78901339955501
C	-2.57905295506371	-3.37153427815369	2.05666187082212
C	-1.78630516562163	-3.59670723217852	0.93230256808367
H	3.94580157098270	3.67288768238447	-2.35662753464090
H	5.38610777423331	-1.38727750797955	2.31211874680536
H	-5.34294265240100	1.56627022860814	-2.27135360857251
H	-1.88489559426419	4.70168732613373	2.98742036997731
H	1.02691367640441	-4.75569276299822	-3.17624530934658
H	-3.15799785048543	-4.18822130441616	2.49325671311316
H	4.60129535633671	2.00829390531170	-0.62634697681807
H	-0.08965416041662	2.16180730329641	-2.08629644265845
H	1.53266277067580	3.76281156457485	-3.10629730535840
H	5.07161875475791	0.34326176528576	0.55088843756974
H	3.35106506552335	-2.49726266869802	3.31296458936920
H	-4.32783355649380	2.68398184297381	-0.29039804236875
H	-1.69884577646498	-0.71714186235391	-2.68797154370405
H	-4.00259732709756	-0.17788910158175	-3.51027277730898
H	-3.16301306296194	3.79501797512532	1.05013501711377
H	0.25588593654089	3.54711009336495	3.67808314878764
H	0.99210224074658	1.53753248045881	2.39146329620434
H	1.08988677756744	-1.80768388539768	2.48571776502656
H	-1.86781405116859	-0.07245328798697	2.42906464737183
H	-3.22087975115432	-1.86705346283531	3.49152950188010
H	-0.48269189977539	-4.76010118026421	-1.19001156683815
H	2.14579323032406	-2.58976586378911	-3.84365944952929
H	1.70437273460309	-0.55099506063705	-2.49116075728074
H	-1.73840385379894	-4.58773028591590	0.48199319462219

==

TS_{a1} (Quartet)

Gibbs free energy = -2868.12452819 Hartree

Co	0.02008598555651	-0.00589841387729	0.00441410720008
C	3.21034698294833	3.14285382618118	-1.99709254507016
C	3.58593517515715	2.23028429973024	-1.01149280858117
C	2.63395243704056	1.32947411780823	-0.51901431754642
N	1.36650977361810	1.33548741692646	-0.98225321952810
C	1.00478517929783	2.21150425178300	-1.92561448229140
C	1.89627251438267	3.13628707769427	-2.46749455101635
C	4.22357766565722	0.10406765179187	1.03301539265190
C	4.41238005078414	-0.86782671540011	2.01535750356259

C	3.31472858114863	-1.60048718913850	2.46981186718193
N	1.88360061637022	-0.39373608981324	0.97819588808166
C	2.93583789821906	0.32144911161183	0.52839890257216
C	-4.23678290897891	1.48704171728781	-1.93893187172215
C	-3.66096373335423	2.11585362783742	-0.83522879302636
C	-2.42221885101388	1.66526311138968	-0.36388309616734
N	-1.78477110203195	0.63521714929213	-0.95861696514944
C	-2.33678535829459	0.03049065733435	-2.01585711173488
C	-3.56475639235096	0.42470677452018	-2.54551109613305
C	-2.19483993395366	3.41965893362620	1.45360635232177
C	-1.47787014634722	3.92040668802444	2.54005402864212
C	-0.31460086435159	3.26606919497711	2.94900618797579
C	0.08664291204326	2.13048936070365	2.24680400720875
N	-0.60372495170056	1.64905048899125	1.20757205524097
C	-1.73019170907412	2.27108194816320	0.80150912231884
C	2.06399413915007	-1.32852138121222	1.91722521607361
C	-1.13941535961023	-2.72810635588948	0.36773034687808
N	-1.14995424082200	-1.51886659634877	0.96669329547070
C	-1.93778852639434	-1.30599932889890	2.02606009775294
C	-2.76352100425723	-2.29725449605797	2.55452312362071
C	-0.01398766122866	-4.07945685035955	-1.46155975219341
C	0.85538327239235	-4.11135101761665	-2.55176050227968
C	1.48524655522636	-2.93341490604429	-2.95675251877453
C	1.21807023258024	-1.76362499480117	-2.24688886709105
N	0.38137320791019	-1.73354738665715	-1.20435754067729
C	-0.23477524287860	-2.86452515126281	-0.80160592424774
C	-2.76478433147340	-3.55236887631807	1.94398445771677
C	-1.94470649615623	-3.77249588333104	0.83752747251810
H	3.94045641079298	3.85354576749678	-2.39095265819875
H	5.41087090829023	-1.05052215061682	2.41901296767329
H	-5.20355157458246	1.82539553237067	-2.31877418881009
H	-1.82630652969311	4.81597739243972	3.05953085171661
H	1.03943005030053	-5.05126159858825	-3.07709075950597
H	-3.40034167857520	-4.35630681597199	2.32215198900125
H	4.60653238559982	2.22832905711869	-0.63041332846726
H	-0.03424711283458	2.16917655033812	-2.26063194026033
H	1.56032418065167	3.83281134414719	-3.23755489798131
H	5.07234302038576	0.67855097548536	0.66365317840294
H	3.41809622293666	-2.37056019012315	3.23618910376817
H	-4.17670058553535	2.94237075779408	-0.34761400907241
H	-1.77487903737774	-0.79670387549358	-2.45599281697806
H	-3.97862467950657	-0.09517689783080	-3.41121679104393
H	-3.09990026439586	3.92400156608318	1.11676245471367
H	0.27680820393043	3.62397825475800	3.79346292497515
H	0.99068226476753	1.58557333061503	2.52876670059975
H	1.17540048737982	-1.87605363917317	2.24046471008237
H	-1.90547650144138	-0.30735382044814	2.46802371449011
H	-3.38954956009792	-2.08191905786593	3.42211362712550
H	-0.50592003486091	-4.99330584749281	-1.13000329325145
H	2.17317632302102	-2.91386290571158	-3.80374807081953
H	1.68852470164035	-0.81742654964715	-2.52473501394653
H	-1.94097496600621	-4.74469495233075	0.34593908402787

===

TS_{a2} (Singlet)

Gibbs free energy = -2373.02641664 Hartree

Co	0.00028709257020	0.00175490208363	-0.00770657085489
N	-1.42920557411403	1.20573843925304	-0.40172189274106
C	-1.29189351260548	2.40532377095264	-1.00069889493313
H	-0.27461352844436	2.78135817331404	-1.11485848588448
C	-2.37885634055167	3.14226282124684	-1.46040477375271
H	-2.21016182329448	4.11090528818177	-1.93432205914245

C	-3.66747137454295	2.62165349938740	-1.30373261164324
H	-4.54168564625774	3.17726563956999	-1.64950328649600
C	-3.81614118087620	1.36671091107517	-0.71421844853914
H	-4.80327436236469	0.91621663611688	-0.60513747111219
C	-2.68023761644289	0.67643879008654	-0.28603978162901
N	-1.42707475455484	-1.20285751890330	0.39313121552414
C	-1.28693824744678	-2.40291320126647	0.99061489771746
H	-0.26943063221297	-2.78020594885931	1.09792612759720
C	-2.37141068156263	-3.13908560075488	1.45734715194313
H	-2.20017411497018	-4.10810138789127	1.92960144341610
C	-3.66065829363302	-2.61737451405297	1.30993456190764
H	-4.53293419121240	-3.17232355998476	1.66160493154462
C	-3.81218907396178	-1.36209764285937	0.72185691706818
H	-4.79964222490697	-0.91068160909626	0.61958524026961
C	-2.67865204637177	-0.67274943237080	0.28602132958679
N	1.42835114356352	1.20467430915242	0.39256047495311
C	2.67926351580272	0.67243910819623	0.28683140777301
C	3.81345755256312	1.35989108926247	0.72388546599345
H	4.80023012805475	0.90667645191413	0.62300701164129
C	3.66352697046713	2.61561293587235	1.31141740370023
H	4.53637991076906	3.16904363104801	1.66404564047797
C	2.37505240812892	3.13969460847571	1.45708675268031
H	2.20504166840058	4.10927070922437	1.92863412144697
C	1.28981411811912	2.40525064118510	0.98945451807666
H	0.27284733298397	2.78433595481639	1.09560513580640
N	1.42754605164555	-1.20396182630778	-0.40246496658428
C	2.67933495768391	-0.67673937612613	-0.28522363665771
C	3.81459891694803	-1.36872987850584	-0.71227595478381
H	4.80236093186945	-0.91992247333472	-0.60193635820711
C	3.66451913853140	-2.62325277268218	-1.30236570504655
H	4.53820133242118	-3.18018424650420	-1.64735513316148
C	2.37522968517821	-3.14159527851460	-1.46079822234395
H	2.20542379634027	-4.10975332061170	-1.93530790487872
C	1.28891600497189	-2.40303255113524	-1.00211796510873
H	0.27114056331487	-2.77728017065332	-1.11774862562366

==

TS_{a2} (Triplet)

Gibbs free energy = -2373.02067690 Hartree

Co	0.00003571671521	0.00020016722706	-0.00025986808984
N	-1.52957323919771	1.18340687583098	-0.57760523610204
C	-1.43068149630779	2.37825033932185	-1.19011616115402
H	-0.41791528182182	2.75544229051823	-1.35594634525198
C	-2.54250574165001	3.10901255027504	-1.58982139710049
H	-2.41254390994110	4.07406219585133	-2.08294681002035
C	-3.81699989096948	2.58117141386719	-1.33716153433130
H	-4.71533379617940	3.12804736823767	-1.63194507320914
C	-3.92518473064622	1.34658604689791	-0.70316845000123
H	-4.90765050681334	0.91986898097257	-0.50117824486342
C	-2.76176905668146	0.65743226129052	-0.33238702600989
N	-1.52992793376470	-1.18249412582866	0.57692777707184
C	-1.43139934494096	-2.37738993322063	1.18941304499351
H	-0.41875981066480	-2.75508154325936	1.35488455910218
C	-2.54343719522720	-3.10760067489263	1.58952352985178
H	-2.41376858136276	-4.07270498497082	2.08262130144342
C	-3.81777137073300	-2.57914084313175	1.33732598135609
H	-4.71626272594009	-3.12557283826507	1.63244909025680
C	-3.92558149263528	-1.34451431838085	0.70334708999641
H	-4.90790965710952	-0.91732293408157	0.50169862561827
C	-2.76196682590039	-0.65593165492718	0.33214081732705
N	1.53003605044523	1.18254914016538	0.57704592241884
C	2.76202258627118	0.65586991071981	0.33213253154273

C	3.92573439949094	1.34435916816818	0.70322418857778
H	4.90800002873666	0.91707721333060	0.50145840793336
C	3.81811177111978	2.57899451466741	1.33720588796984
H	4.71668648503575	3.12535692125953	1.63220729958253
C	2.54384022054692	3.10755794238069	1.58956402596639
H	2.41431400903006	4.07267291286638	2.08267891912355
C	1.43169707772708	2.37744868321172	1.18959050840242
H	0.41910503187483	2.75521124123089	1.35520297378935
N	1.52933082349753	-1.18315817534108	-0.57779771752890
C	2.76163060446532	-0.65745471146801	-0.33242413266064
C	3.92494051781343	-1.34684877730569	-0.70313818334430
H	4.90748163019048	-0.92036402763638	-0.50101675767228
C	3.81655575664579	-2.58135657435583	-1.33723215419242
H	4.71480352794724	-3.12841187209526	-1.63195109316615
C	2.54196215004811	-3.10889441512163	-1.59008823978606
H	2.41184622968251	-4.07386265970493	-2.08332975538632
C	1.43025855077913	-2.37792699663263	-1.19045089839756
H	0.41742742042380	-2.75487007767098	-1.35646440405579

==

TS_{a3} (Singlet)

Gibbs free energy = -2561.61286681 Hartree

Co	-0.00121005563625	-0.00009422303889	-0.20037887993354
N	-1.78813265147177	-0.73253748479547	0.01330064548759
C	-2.06857440967129	-1.93718935069821	0.53607637112746
H	-1.22152721726233	-2.58955871497650	0.74407178044884
C	-3.36852995145966	-2.33555194868515	0.83500966565731
H	-3.54522442683152	-3.32856522406156	1.25123209735571
C	-4.41520055806773	-1.44176050719347	0.60366153692628
H	-5.44749099755304	-1.71938451693582	0.82754588224611
C	-4.12281552370190	-0.17455122992753	0.10028803804507
H	-4.91889913542605	0.55251408351819	-0.05997613392816
C	-2.79462235633768	0.15740309717122	-0.17549335821552
N	-0.99978867352042	1.61540136057994	-0.70573778921688
C	-0.48367392020855	2.72463872684527	-1.25585863771605
H	0.60207038520232	2.78964504647016	-1.31972342252235
C	-1.27884125946838	3.76297989971831	-1.73755871343819
H	-0.80643552761605	4.64717767566914	-2.16873340595588
C	-2.66594459570634	3.64031681776737	-1.65638668717923
H	-3.32101214282013	4.43618503751702	-2.01707048240547
C	-3.20788622255779	2.46887824209069	-1.12776768929193
H	-4.28790818858303	2.32967528543608	-1.08777049449284
C	-2.34816625672828	1.46620649927608	-0.67481871175867
N	1.00338149840724	-1.61574340771454	-0.71173492413743
C	2.35109254047335	-1.46222570241603	-0.68086476297523
C	3.21379027939753	-2.45884151396981	-1.14153798938152
H	4.29338483839122	-2.31634174524132	-1.10200048451034
C	2.67522561619432	-3.62849962052044	-1.67761464269313
H	3.33267183727995	-4.41952645015189	-2.04458514579987
C	1.28848601187419	-3.75566300848823	-1.75783668185561
H	0.81869791674419	-4.63872489599084	-2.19416649338634
C	0.49046555437161	-2.72317568182917	-1.26823950983681
H	-0.59519519126653	-2.79171020476256	-1.33073852847152
N	1.78644344007925	0.73195231796828	0.02072379017977
C	2.79422797206545	-0.15565400657019	-0.17197965139036
C	4.12151202953438	0.17567849334517	0.10896856288334
H	4.91858321598757	-0.54959275459465	-0.05442283473447
C	4.41179331792393	1.43959078793630	0.62176687084463
H	5.44335008558119	1.71642004910509	0.84997637608030
C	3.36384860614556	2.33080001319974	0.85687854104383
H	3.53863812584427	3.32104364404894	1.28043003874219
C	2.06495482205200	1.93332873120090	0.55216923539586

H	1.21701219148006	2.58367493907932	0.76276316688547
C	-0.01113001525556	0.00923517827862	1.82147414633061
O	-0.45622937979403	1.06714280021658	2.21397595168010
O	0.43273837191474	-1.05086053387613	2.21038835786692

==

TS_{a3} (Triplet)

Gibbs free energy = -2561.62508904 Hartree

Co	-0.00102690732591	-0.00082624101231	0.58814375403498
N	-1.89631710283435	-0.69710818217418	0.62408974278509
C	-2.29862745496499	-1.80810332165867	1.25682707035148
H	-1.54248800250277	-2.32598982754625	1.85078256064388
C	-3.60700022608205	-2.28037714045041	1.17513302455499
H	-3.89097999764463	-3.18937847266401	1.70816733284068
C	-4.52613847382156	-1.56509807085376	0.40614813274468
H	-5.56167814195962	-1.90096945238558	0.31705011073198
C	-4.10566554914862	-0.41132368735345	-0.25552322603974
H	-4.80776627994951	0.15368031304868	-0.86798101933797
C	-2.77564298660193	0.00295941535541	-0.13049562354215
N	-0.90102028296216	1.40303990964345	-0.64625997547492
C	-0.30584926387613	2.44297338541232	-1.23914315837573
H	0.76891741978560	2.55234150300345	-1.07455341720458
C	-1.00736936577180	3.35049374259493	-2.03229307464701
H	-0.48382781334505	4.18688658743226	-2.49877233333558
C	-2.37898519300488	3.15749422922602	-2.20618365913742
H	-2.96621603231729	3.84774193910334	-2.81620095456712
C	-2.99803618687320	2.06826596123643	-1.59160282518278
H	-4.06765111154305	1.90383213972213	-1.71848760639612
C	-2.22582320536845	1.19938951151686	-0.81319973627971
N	0.90213427790038	-1.39800175683400	-0.65090508604997
C	2.22722993200524	-1.19320213594453	-0.81403841182915
C	3.00147414158321	-2.05860248079164	-1.59428559792803
H	4.07134263983507	-1.89339297049377	-1.71796946375009
C	2.38409435822073	-3.14534989690940	-2.21491738618820
H	2.97291149880593	-3.83287565147808	-2.82646909875821
C	1.01212181693331	-3.33941605305932	-2.04503899820940
H	0.48986750521104	-4.17393148289541	-2.51629074879357
C	0.30855161058159	-2.43550182302305	-1.24958518207681
H	-0.76655376857651	-2.54587656887739	-1.08775853364539
N	1.89368842305508	0.69686957720355	0.63066668101136
C	2.77497300288434	0.00064923146300	-0.12519614162501
C	4.10491532015665	0.41648718833205	-0.24592432201411
H	4.80863436379313	-0.14532875490544	-0.85947454825724
C	4.52321484435666	1.56775881408530	0.42147954049254
H	5.55865929908077	1.90481950700636	0.33578658097469
C	3.60207005158222	2.27905241439445	1.19177664855534
H	3.88434256061980	3.18596041267942	1.72925876071446
C	2.29391438330200	1.80542168119452	1.26895799384496
H	1.53631446620136	2.32003625947180	1.86391262920472
C	0.00070284000798	-0.00881129015418	2.55795409152911
O	-0.34045999266023	1.09739712897029	2.90827983236268
O	0.34297858323257	-1.11994959063122	2.89184064126836

==

TS_{a4} (Doublet)

Gibbs free energy = -2561.74689532 Hartree

Co	0.02480773040950	-0.08926871006761	0.80063836713735
N	-1.86547873679759	-0.74785392495005	0.74572710203442
C	-2.31876801264112	-1.80536083768060	1.44128376945774
H	-1.60784942133470	-2.23714389419095	2.14880664877657
C	-3.60753802024394	-2.30289089952562	1.29103331707865
H	-3.93055992650417	-3.16625409560003	1.87603249743998
C	-4.46986430409529	-1.67139286476504	0.38321459259120

H	-5.49053292520154	-2.03251359676367	0.23821421412832
C	-4.00558239690933	-0.57247761968115	-0.33260733540113
H	-4.65934258409248	-0.06525325666393	-1.04237221877061
C	-2.68902461453666	-0.12662221916264	-0.13565452194263
N	-0.80170347654059	1.28079414286077	-0.51466291902987
C	-0.18062178097263	2.31720471938016	-1.10007583134358
H	0.86136942658704	2.48263835925907	-0.81467160528525
C	-0.80498516319390	3.14948016609352	-2.02247210034238
H	-0.25419087035504	3.98005950966776	-2.46856669361050
C	-2.14178041027051	2.89345744456509	-2.35995156807872
H	-2.66763156313669	3.52387027213593	-3.08056475957595
C	-2.79047476478178	1.81876429227021	-1.75993321602206
H	-3.82996525114375	1.59978920662478	-2.00559571974889
C	-2.09446676069383	1.02362853868510	-0.83555976230199
N	0.82342004132516	-1.31314540775069	-0.60100212257595
C	2.12990200719373	-1.08163057956674	-0.88625315649672
C	2.82135350100934	-1.85864442618580	-1.82543537309914
H	3.87198437037299	-1.66191671830652	-2.03970754656067
C	2.15331068463398	-2.88941623376736	-2.48195058991479
H	2.67605131785770	-3.50613391956993	-3.21640784664419
C	0.80465660863357	-3.11787364798912	-2.18320330083442
H	0.23905803561994	-3.91186453294278	-2.67471116362363
C	0.18338861732115	-2.30686013840174	-1.23890390803703
H	-0.86797165545695	-2.45318424418323	-0.98067942669286
N	1.90877380894264	0.62772095595160	0.76360526853444
C	2.73252265317031	0.03561544190469	-0.13877937896882
C	4.04513872099060	0.48677945330887	-0.33491558236513
H	4.69308762771167	-0.00043140762881	-1.06356844700767
C	4.51171794528289	1.57142660913403	0.40293361543353
H	5.53183146464723	1.93586978508961	0.26261550062720
C	3.64971646047342	2.18642103808992	1.31729386286804
H	3.96840224685652	3.04427114799674	1.91249839085759
C	2.36025342654696	1.68336369525247	1.46398550855578
H	1.64868648921313	2.12756139383706	2.16338796377558
C	-0.19918032329458	0.43650467710300	2.60230801858301
O	-0.44247456224846	1.50088248021165	3.18168746024820
O	0.08848433964610	-0.71383415407804	3.03064499614697

==

TS_{a4} (Quartet)

Gibbs free energy = -2561.73707863 Hartree

Co	-0.03048166960402	-0.04987638900337	0.52474909936962
N	-1.88614014690345	-0.77457379226581	0.52113315217739
C	-2.32089119783562	-1.87663787006533	1.16276298433205
H	-1.54467707538859	-2.47333393934883	1.65000696203253
C	-3.65232455101685	-2.24934558862610	1.22304064067286
H	-3.94418728755074	-3.16443392669439	1.74095833398874
C	-4.61181429391620	-1.40288522184027	0.60380264559495
H	-5.67627686594216	-1.64684050816183	0.64696147342723
C	-4.18637373791746	-0.26300953869650	-0.04633094335965
H	-4.91369628629385	0.40211810983627	-0.51435301976072
C	-2.80195465529935	0.05378049858211	-0.10694700110449
N	-0.88980112970883	1.37532801086286	-0.65900931537661
C	-0.29335047029244	2.39219550772718	-1.30391875283728
H	0.78807474637482	2.48070327255747	-1.16140700989322
C	-0.97387673329594	3.29673655324693	-2.10307774585647
H	-0.43806932769203	4.10944191397998	-2.59631968559340
C	-2.37647548984369	3.12900876871687	-2.25021729063867
H	-2.95413418503703	3.81640116082907	-2.87357437731258
C	-3.00485634715428	2.08211777781122	-1.60677995413269
H	-4.07906621862122	1.93375873184619	-1.72659745178122
C	-2.25188325666244	1.18516263307389	-0.80101760749930

N	0.96343825707444	-1.44124804694761	-0.67979332864769
C	2.28359547240626	-1.20018425771676	-0.83226833661788
C	3.08267439164839	-2.03353927531094	-1.62531627412108
H	4.14731816941734	-1.83603888435322	-1.74784017144971
C	2.49584906704676	-3.12597172909281	-2.26300803933893
H	3.10350017353126	-3.78780375373448	-2.88439934149351
C	1.12708434090706	-3.35931434088265	-2.09989312469589
H	0.62979798832827	-4.20208256525290	-2.58334170761634
C	0.39801475568293	-2.48360234993258	-1.29687089301477
H	-0.67554439512068	-2.61523347501937	-1.13814771085881
N	1.86116752428829	0.68646123069707	0.58200214274399
C	2.78886800866584	-0.00433251405839	-0.12143151517445
C	4.12132774904334	0.42414693612760	-0.17213964213466
H	4.86518067861846	-0.13856095823170	-0.73603802656668
C	4.48964973054407	1.58661684017268	0.50285589417098
H	5.52450367461065	1.93461628575598	0.47114703915589
C	3.51824978180220	2.29626166707105	1.21456582796802
H	3.76099897172108	3.21320890607789	1.75436235076035
C	2.21525080514020	1.80495505308818	1.22984475650098
H	1.42010335852557	2.30783137441962	1.78488963129393
C	-0.02477476180104	-0.09688179772419	2.46877836267073
O	-0.38901075915953	0.98816134504225	2.89412089150007
O	0.34294319668018	-1.19914585456233	2.84176107851639

==

TS_{a5} (Doublet)

Gibbs free energy = -2562.20938596 Hartree

Co	0.10571825639743	-0.07409019970929	-0.80139426499695
N	1.95678276344199	-0.69665893200470	-0.58591044583111
C	2.46790080066781	-1.84812358036301	-1.05451788003681
H	1.79229233555348	-2.49037370272982	-1.61923109659389
C	3.78867793338887	-2.22932830863278	-0.83100939856540
H	4.15236866915819	-3.17410108875478	-1.23857152941451
C	4.61514911712015	-1.38592492543436	-0.08784533109733
H	5.65786550133044	-1.64844249175306	0.10354893981713
C	4.08530717511987	-0.19989754274957	0.41710357757446
H	4.70593469146731	0.46919342496440	1.01218551025736
C	2.74914303847120	0.11892009319654	0.15810156489943
N	0.77470055915005	1.43545785017658	0.34747140760585
C	0.07062184805142	2.48777588166814	0.78401357876558
H	-0.98123348672834	2.53191613719587	0.49724159789924
C	0.63546702341889	3.49210283843046	1.56795899876703
H	0.02104889315551	4.33078675314063	1.89976781632986
C	1.98570067253767	3.39512801753998	1.90819509136215
H	2.46507340346183	4.16354568129776	2.51870157331350
C	2.72004284343483	2.29860178612391	1.45799184297293
H	3.77476699776148	2.20201997454993	1.71434668521930
C	2.08353829242886	1.32899494015481	0.67635428800532
N	-0.82112626939289	-1.03717598763364	0.82276331063196
C	-2.10055273001631	-0.63645343947308	0.98000762286043
C	-2.86378381509845	-1.05335481446634	2.07681725697379
H	-3.89416374007209	-0.72033989780608	2.20040430972605
C	-2.28148335651651	-1.89907854025676	3.02107353822254
H	-2.85910931050162	-2.23542043926923	3.88503034728289
C	-0.95570299054579	-2.30291495908017	2.84905573343900
H	-0.46215272489474	-2.96106927628194	3.56652120805947
C	-0.26097187056513	-1.84228378739101	1.73041613290446
H	0.77949538140736	-2.12931129120468	1.55438515647169
N	-1.70007622715702	0.63155499707160	-1.01430113400086
C	-2.60764758420104	0.26713395371167	-0.07779067297297
C	-3.92481195768164	0.73480103472342	-0.13991735362299
H	-4.65380566552700	0.43458322933282	0.61232927787158

C	-4.29837924292789	1.58545648088962	-1.17954772495500
H	-5.32278528710131	1.95945511315906	-1.24000434961482
C	-3.35013102436361	1.94535325227773	-2.13988408339629
H	-3.60185042305271	2.60402887238301	-2.97276112634648
C	-2.05848909856173	1.43864787563369	-2.02225628729726
H	-1.28426975798079	1.66877787274685	-2.75721616141559
C	-0.29490905276275	-1.24857059396972	-2.24466299950776
O	-0.23178338103208	-0.85513943808471	-3.39716058329398
O	-0.66540662721806	-2.55456312561839	-2.05928807918928
H	-0.71421557302514	-2.72620269770135	-1.10515586508372

==

TS_{a5} (Quartet)

Gibbs free energy = -2562.19423732 Hartree

Co	0.07873613266712	-0.28657945286642	-0.71636519136096
N	2.07363353678724	-0.86580315163401	-0.35539052294116
C	2.60249971859335	-2.02205606216168	-0.77227732396130
H	1.92362792370829	-2.71338243411131	-1.27882003465511
C	3.94797590733813	-2.33391708919822	-0.58632681178931
H	4.34145692926784	-3.29001125790993	-0.93571936369646
C	4.76332268658341	-1.39314061185570	0.04577315335912
H	5.82499161703347	-1.59450712502137	0.20561071609582
C	4.21207760596101	-0.18246411524129	0.46628132476017
H	4.84203555710619	0.56626495436075	0.94520017442581
C	2.84930956648895	0.05554140165547	0.25166852803488
N	0.88886411411777	1.46001256001280	0.23239300916781
C	0.21092443580834	2.56710462782041	0.55625078664344
H	-0.81810105640839	2.63322224191892	0.19958472818747
C	0.77092387516859	3.59783944726374	1.30972894803969
H	0.17912228897346	4.48384130964527	1.54585151745970
C	2.08800892915495	3.45870053047593	1.74788322647920
H	2.56410908041493	4.23925799351346	2.34564601059225
C	2.79457210477856	2.30268584675581	1.41661705123737
H	3.82072460469529	2.17402369452092	1.75915623156754
C	2.16653393648693	1.31367378830563	0.65097288898747
N	-0.96903509061181	-1.08360389890139	0.89058695695286
C	-2.23917375254099	-0.66091270799811	1.08086007651533
C	-2.97910658956730	-1.08456043770722	2.19077164361084
H	-3.99882167020477	-0.73309667601845	2.34552500231793
C	-2.39294916180565	-1.95808270655458	3.10607858397739
H	-2.95728693767903	-2.29697231200256	3.97782707829164
C	-1.08083267883253	-2.38726726485732	2.89592479849414
H	-0.58389302445962	-3.06904837049586	3.58815252421590
C	-0.40368103451394	-1.91890626510272	1.77221414703190
H	0.62722343484129	-2.21955970685727	1.57024563102351
N	-1.88234295106185	0.64038425859846	-0.89097564650034
C	-2.77035902223281	0.25794074045884	0.04661657887485
C	-4.09824794204293	0.70343485880775	0.01360951482450
H	-4.81615250750485	0.38204981516981	0.76789939482622
C	-4.49459648438179	1.56338639076200	-1.01030900702845
H	-5.52461069702260	1.92474779155520	-1.05327688950008
C	-3.56389915277158	1.94698412811679	-1.97907364040245
H	-3.83658188721310	2.61324954555840	-2.79951316494529
C	-2.26378722841553	1.45185469919484	-1.88221812760102
H	-1.50044520149462	1.69304752341428	-2.62632305633430
C	-0.17572329975865	-0.98886870677575	-2.62933784796771
O	-0.06343955499690	-0.25239009566565	-3.59761995261982
O	-0.42824869142807	-2.31381440771730	-2.92442586127547
H	-0.53060336902582	-2.80488629123137	-2.09359778341553

==

TS_{a6} (Doublet)

Gibbs free energy = -2486.22114767 Hartree

Co	-0.06743201694221	-0.09704326766579	0.90215991210468
N	-1.93021543778159	-0.67348234219180	0.82470827246348
C	-2.45105254785462	-1.76528091592253	1.41080384933094
H	-1.78517894651455	-2.38324291395638	2.01379693280321
C	-3.78929921781757	-2.11992521985645	1.26428623373794
H	-4.16440869380294	-3.01578989119960	1.76135537273603
C	-4.61630191331369	-1.31360103677405	0.48269855959027
H	-5.67134694205613	-1.56080185925220	0.34711378090526
C	-4.07686278304073	-0.18287747008794	-0.12933269229764
H	-4.70155776830597	0.45852554907256	-0.75001503187481
C	-2.72620163612287	0.11533596068688	0.05693920986568
N	-0.72315086694062	1.35981274189257	-0.26081207772160
C	-0.00133873834006	2.36347974299896	-0.78024644271042
H	1.05732507161535	2.40609355132424	-0.52964206979590
C	-0.56408056746618	3.32928742842524	-1.61169433671169
H	0.06433564563977	4.12811288519731	-2.00833730144571
C	-1.92276410802174	3.24704641088954	-1.91347430633891
H	-2.39769002896477	3.98772486180701	-2.56042249666627
C	-2.67332262697173	2.20049209515916	-1.37809244233750
H	-3.73597275682542	2.11379228721535	-1.60237872738743
C	-2.04504612540379	1.26646586653792	-0.55314452212497
N	0.72203372322619	-1.22324764166408	-0.65562737467883
C	2.00671137974305	-0.89804118996275	-0.91684199283968
C	2.70456000037015	-1.49323037109579	-1.97140258205163
H	3.74121243402468	-1.22781622282752	-2.17731298033792
C	2.04910670810335	-2.43779327979769	-2.76270644242730
H	2.57508826211054	-2.91472865814974	-3.59267841702856
C	0.72125905989016	-2.76144267489162	-2.48152617138946
H	0.17357882862474	-3.49409041589485	-3.07678521169749
C	0.09159982790850	-2.12597216464343	-1.41037221259184
H	-0.94668668351950	-2.34954865849137	-1.15452083372483
N	1.74608083379750	0.60271988045772	0.93322741810546
C	2.58670518945580	0.11703890366584	-0.01282574111393
C	3.90085635442819	0.58544009271364	-0.09101766689978
H	4.57811280319545	0.20173752990895	-0.85320961361211
C	4.33777285887461	1.54938956738212	0.81737826425429
H	5.36294443582859	1.92242130513683	0.76607447978271
C	3.45624975706786	2.02816255715958	1.78884678119952
H	3.76000127990507	2.77980466961669	2.51892075151394
C	2.15937242154351	1.52436545346392	1.81564615049925
H	1.42873967626328	1.86652362595703	2.55260181796577
C	0.44196342507552	-1.24251828905567	2.26804480061658
O	0.82707042931480	-1.92938548328781	3.08003310033119

==

TS_{a6} (Quartet)

Gibbs free energy = -2486.21102456 Hartree

Co	-0.09430190740950	-0.26229668608853	0.60590152263696
N	-2.10375849416370	-0.74355832073972	0.63379850972623
C	-2.62366451002940	-1.86003200972934	1.16332094179269
H	-1.92207373514501	-2.57516858631521	1.60123799318819
C	-3.99276832013757	-2.11522538815625	1.15934233460961
H	-4.37665305510006	-3.03700755389353	1.59917461466511
C	-4.84117600995919	-1.16697292565865	0.58639532010499
H	-5.92141906424880	-1.32702731568978	0.56601539214467
C	-4.29819115803267	-0.00535601761573	0.03559044522844
H	-4.95126081910549	0.74152588397064	-0.41364667874961
C	-2.91383269606254	0.18282650660717	0.07063529435246
N	-0.89037375449716	1.41035685031933	-0.36583008612204
C	-0.19271190115013	2.42985827072448	-0.88200006964305
H	0.89054666970292	2.40094742633400	-0.75568115657263
C	-0.80688655637552	3.48616383906539	-1.55177439427210

H	-0.20175610952944	4.29868066481246	-1.95695329798008
C	-2.19537050236375	3.46958297643190	-1.68535313271658
H	-2.71561648853081	4.27975115006322	-2.20118468601683
C	-2.92033293437849	2.40051700582037	-1.15674225717638
H	-4.00398346885680	2.37150100288081	-1.26261376362751
C	-2.23664608686849	1.37348764493776	-0.50031533176097
N	0.93538965616924	-1.32601439398337	-0.79570051544013
C	2.24896892044251	-1.03553208718985	-0.94106450341552
C	3.02821684191484	-1.71355353083447	-1.88258809306263
H	4.08645811629532	-1.48249319871311	-2.00007712496090
C	2.43271980192369	-2.69476444821968	-2.67631769708253
H	3.02918297208355	-3.23340349934971	-3.41601234855393
C	1.07437160419616	-2.97538956193553	-2.51851228207567
H	0.57198235889533	-3.73190908120539	-3.12333449486360
C	0.35714858551187	-2.26118203847212	-1.56167500055219
H	-0.70895080000506	-2.44093007010287	-1.40232826345124
N	1.85674221072677	0.57739161684560	0.78367256376383
C	2.76533769116264	0.03353213438641	-0.05617283956104
C	4.08998426107516	0.48116955535097	-0.07088832832326
H	4.81961481754067	0.04295423279793	-0.75077139302511
C	4.46780326483750	1.50479242714203	0.79872349375741
H	5.49794132383593	1.86778902006009	0.79991343391128
C	3.51867586372306	2.05570292838247	1.66152734416883
H	3.77428663533600	2.85751704898018	2.35610814106556
C	2.21781114955842	1.55721403139352	1.62153293485555
H	1.43960673410946	1.95676138585887	2.27807577855927
C	0.45130701913060	-1.19609111416846	2.51702984633223
O	0.86040187377795	-1.65220277510430	3.45975783414225

==

TS_{b3} (Singlet)

Gibbs free energy = -2373.43206518 Hartree

Co	-0.01121029841393	0.01627788608895	-0.91562884368149
N	-1.82293940947445	0.67866499231909	-0.89318404078419
C	-2.28235745932603	1.66750689677213	-1.67919556981140
H	-1.55340958991907	2.16133559041926	-2.32263881786063
C	-3.62050176753553	2.05132140825397	-1.66625970676476
H	-3.95609776583724	2.85881832249626	-2.31853758649750
C	-4.49957877684708	1.38832119002817	-0.80946244905830
H	-5.55506326304495	1.66641454622509	-0.77128510340509
C	-4.01966935183549	0.36062835217512	0.00426708718801
H	-4.69328962547960	-0.16943858131582	0.67692921621767
C	-2.66888394020811	0.01889851535514	-0.05739641845104
N	-0.72766350407314	-1.24953139385575	0.49016415904207
C	-0.04781694489827	-2.18088652838596	1.16840201027692
H	1.01063002513365	-2.29869804677397	0.92841693619147
C	-0.65300824751317	-2.97357980596995	2.14301242319829
H	-0.06440023557826	-3.72350529443174	2.67371957345090
C	-2.00777927193829	-2.78105146410719	2.41344835532430
H	-2.51539740259832	-3.38293852905952	3.17037190313736
C	-2.71499378542600	-1.80477327646677	1.70833381651999
H	-3.77256161412054	-1.63758288204635	1.91009404425393
C	-2.04213648525655	-1.04809135236973	0.74832273892139
N	0.83914431970512	1.24397112895209	0.21245870242931
C	2.17113674395133	1.02713732895020	0.37776375722501
C	2.92670633092051	1.84710050492054	1.21492684498798
H	3.99351327331238	1.66279386868865	1.34121698771253
C	2.29790578462512	2.89575854693875	1.88579716886638
H	2.87420202317494	3.54884917000560	2.54436778917036
C	0.92902287031480	3.09634778740737	1.70575893586069
H	0.39594135708958	3.90274020217680	2.21159565344414
C	0.22541977248857	2.24621011418541	0.85592195811423

H	-0.84262253105507	2.37411397822627	0.68828774102819
N	1.72050257716609	-0.75852772803453	-1.08196354793155
C	2.68457845364535	-0.11735829550613	-0.38191630203825
C	4.00713827762633	-0.55894633366198	-0.41603096741287
H	4.78018876605458	-0.04017813925702	0.15095820768461
C	4.31891501751248	-1.67730625797379	-1.19114778008508
H	5.34774453316158	-2.04087931106440	-1.23439290199439
C	3.30901336188729	-2.32487116587390	-1.90682848444563
H	3.51702954243718	-3.20214361848511	-2.52109131386308
C	2.00775532516141	-1.83258102916684	-1.82698420137770
H	1.17962010636769	-2.30448557864980	-2.36347833623164
H	0.36172380864308	0.90410828187140	-1.99578963855113

==

TS_{b3} (Triplet)

Gibbs free energy = -2373.43334618 Hartree

Co	-0.05766777230930	0.03132774669186	-0.87478074557158
N	-1.88538275796364	0.65157608055918	-0.90746038864196
C	-2.33062898389107	1.67779372743983	-1.64955785405723
H	-1.60258896733518	2.17420599784372	-2.29354437373237
C	-3.65937500906509	2.09015567295838	-1.59824867618669
H	-3.98820619167537	2.92889821833971	-2.21354198810595
C	-4.53999139829395	1.40771363788102	-0.75804874612058
H	-5.58981535042904	1.70235827044232	-0.69619914182529
C	-4.07005031610493	0.34034028192096	0.00921740153964
H	-4.74447302158444	-0.19847564662249	0.67395338776151
C	-2.72592857475782	-0.02260657307793	-0.08064951269686
N	-0.77871261652408	-1.29725413152081	0.46641455401813
C	-0.10193572492654	-2.24654032197896	1.12440626584345
H	0.96469016504366	-2.33692336137649	0.91074925728730
C	-0.72443254858545	-3.08543501742523	2.04675701664673
H	-0.14192804316976	-3.84908900707775	2.56438332579509
C	-2.08982129413159	-2.92105832409692	2.28300723252908
H	-2.61138122624717	-3.56248660621639	2.99679297741963
C	-2.79101754630948	-1.92442392014585	1.60034046143465
H	-3.85690271166490	-1.78246168992456	1.77646440146401
C	-2.10415841274750	-1.11851703162606	0.69210127404732
N	0.90464450524827	1.36898067430529	0.29618436984399
C	2.23280102225037	1.13045122927969	0.40311936896728
C	3.04939588456428	1.95658945194070	1.17600340869932
H	4.11870766115940	1.76538423787390	1.25935580288913
C	2.47091291409698	3.03820011914833	1.84395722070392
H	3.09301963794061	3.69721875230985	2.45365618161992
C	1.09864429193976	3.26728253543957	1.72672055365818
H	0.61277021977986	4.10068975113407	2.23653720865033
C	0.34400561583296	2.40264097466275	0.93646576460145
H	-0.73291467981890	2.53823768917909	0.81450224253533
N	1.73577553400460	-0.70360945332624	-1.04063302804882
C	2.70097059800803	-0.05031062896730	-0.34778155545120
C	4.01954448346130	-0.50933358107586	-0.36194094298903
H	4.79329934803196	0.01534206615649	0.19822527715603
C	4.33284884122912	-1.65302089963113	-1.09705467869030
H	5.35964772547558	-2.02437402996977	-1.11786283267346
C	3.32411820240101	-2.31301490497132	-1.80081491985206
H	3.52788872670671	-3.20938198963435	-2.38844836948010
C	2.02969825154740	-1.80236589830646	-1.75160088417718
H	1.20753378046731	-2.27761176208899	-2.29036980125691
H	0.03284673834603	0.11287166355418	-2.34945251555383

==

TS_{b4} (Singlet)

Gibbs free energy = -2867.94861063 Hartree

Co	0.01517305331819	0.00328660946128	-0.00195318735158

C	2.99240926777419	3.00618982561175	-2.07706998732955
C	3.38754456248924	2.12315615910415	-1.07263909962313
C	2.44547559464258	1.25026485515154	-0.52837113557723
N	1.16052452664266	1.24957543317273	-0.96264602591968
C	0.77567880940888	2.09737081779964	-1.92673697613613
C	1.66755435094343	2.99280219806999	-2.51168975155051
C	3.99672894651711	0.05231351063067	1.07564593722811
C	4.14320871858319	-0.90575073588837	2.07823464323828
C	3.02374763433419	-1.61902829743100	2.50569561596762
N	1.65364280578950	-0.42917656174386	0.95541879376786
C	2.73310295490018	0.27116289301564	0.52673143722392
C	-4.01546534693306	1.37189245297145	-2.02440383145885
C	-3.48394105042183	1.99869663227205	-0.89789684996101
C	-2.27309671625082	1.54307660787136	-0.37634262551316
N	-1.60914165104971	0.50952646370458	-0.95155931819642
C	-2.11674883800921	-0.09673608797984	-2.03397134276287
C	-3.32209688698414	0.30856102311302	-2.60161460297211
C	-2.08152588186184	3.21840676551046	1.51311329360909
C	-1.35542523969059	3.67811941250005	2.61133401629151
C	-0.17520342121157	3.02599225093911	2.96681428391047
C	0.24149481281892	1.92973771371075	2.21573732711909
N	-0.46215362707941	1.48877575903870	1.16342639927625
C	-1.60705277459179	2.11808888693788	0.79874284390465
C	1.78975700820093	-1.35281210142286	1.91742468365076
C	-1.07201653896122	-2.53019257850542	0.38097443489385
N	-1.07374736669583	-1.30010209718097	0.95262748287453
C	-1.82377324116138	-1.06405821077829	2.03825899590323
C	-2.61374367939567	-2.05728690933601	2.61179258050679
C	0.00403557981166	-3.84203100740545	-1.49983057297617
C	0.85995863387788	-3.83633478804397	-2.60075963455645
C	1.48753567354582	-2.64633681725055	-2.96763327986587
C	1.23829469412334	-1.49603996309005	-2.22314639556190
N	0.41247423352017	-1.50585705086246	-1.16749316239901
C	-0.20148036321244	-2.65629185584132	-0.79426598311486
C	-2.62377067086024	-3.32727470408893	2.03608307556785
C	-1.84245359942215	-3.56770494682226	0.90642794682752
H	3.71609187137660	3.69745683519295	-2.51379883370929
H	5.12475245527972	-1.09104057982213	2.51926788069961
H	-4.96293566244800	1.71416319670843	-2.44580955743478
H	-1.71118232127787	4.53890581208969	3.18121403812114
H	1.03411631997018	-4.75558775265418	-3.16371954689938
H	-3.23372969595308	-4.12757969234846	2.46007096834770
H	4.41644324691194	2.11598061743485	-0.71487925483646
H	-0.26247140805044	2.05968634110367	-2.25143729095596
H	1.31348258386511	3.66340430275348	-3.29578120922927
H	4.85598778222169	0.62131759840774	0.72245752191117
H	3.09021763019151	-2.37660129526375	3.28773921974509
H	-4.00694014782231	2.83218691489320	-0.43029647178289
H	-1.54825871437439	-0.91639450009670	-2.46948721022035
H	-3.69791604538633	-0.21080053087102	-3.48419980052276
H	-3.00544967837111	3.71196547288520	1.21371316103608
H	0.42584161942280	3.35025681593055	3.81737653420984
H	1.15294130457883	1.39601620095743	2.47875895554519
H	0.89765978700187	-1.88813582086660	2.23792447379041
H	-1.79187825695193	-0.06585325580085	2.47104623911379
H	-3.20660223924433	-1.82336159155439	3.49704658900374
H	-0.49562201528682	-4.75972179113282	-1.19165855460945
H	2.16502198860482	-2.59357852384438	-3.82097127904639
H	1.70803576307030	-0.55165393802754	-2.49189725373335
H	-1.83465413477797	-4.55119439298957	0.43777765252167

==

TS_{b4} (Triplet)
Gibbs free energy = -2867.90602102 Hartree

Co	-0.04899069151821	-0.01820710153025	-0.00445694884790
C	3.08100384527967	3.13136307657048	-1.97140061990944
C	3.46826655131445	2.20216116129376	-1.00628275493818
C	2.52256206121172	1.29859199661086	-0.51392456716185
N	1.24946137758028	1.31620463422948	-0.96521029415439
C	0.87059002788923	2.20839050516714	-1.88917602963572
C	1.76108638702833	3.13670014697636	-2.42319624912460
C	4.10765338508632	0.03983430353958	1.01039052672786
C	4.28822435896671	-0.95046547875236	1.97549010767093
C	3.18496760403713	-1.67940568008647	2.42017087256078
N	1.76671609367539	-0.43598801485782	0.95567981770163
C	2.82417471489596	0.27730601941251	0.51110923977368
C	-4.06292650833718	1.49091740656097	-2.15254265498610
C	-3.52327610121818	2.10492546184537	-1.02117477821580
C	-2.33801194994765	1.60389846744856	-0.48324521306003
N	-1.71011056395290	0.54228289337298	-1.04746106225923
C	-2.22713537047150	-0.05312794641152	-2.13092465354690
C	-3.40869500476311	0.39485090903197	-2.71672049837790
C	-2.11796724178103	3.26871331850037	1.41376853606194
C	-1.40665111692064	3.70039832111425	2.53288481255062
C	-0.26254423146820	3.00539012070235	2.92378557951480
C	0.13681158415714	1.89766048022501	2.18153246558734
N	-0.55417926171352	1.48821659076792	1.10762639685873
C	-1.67021266957202	2.15199553085841	0.70682753840972
C	1.93410562633222	-1.39062776692820	1.87975418949092
C	-1.09441306898440	-2.61980761702227	0.48706127170941
N	-1.13683669959960	-1.38542129179430	1.04726761391264
C	-1.88987170301335	-1.15962858284677	2.13203687596775
C	-2.64266364995389	-2.17158168340053	2.72283779999460
C	-0.00276782616498	-3.91054256606976	-1.39936149255222
C	0.82752937799314	-3.89367470832354	-2.51961101425557
C	1.40504747736626	-2.68983505699402	-2.92204666505120
C	1.13646285463555	-1.53761906362938	-2.18839158448869
N	0.33665398648374	-1.56269347932998	-1.11261476777931
C	-0.23721942188817	-2.72452155960990	-0.70304592675273
C	-2.60661048446193	-3.44854067983702	2.16107273621466
C	-1.82374138472824	-3.67842865478136	1.02850459220869
H	3.80752379085202	3.84590221244257	-2.36417525382307
H	5.28535706739927	-1.14959946354810	2.37399627272644
H	-4.99045983334715	1.86880437798597	-2.58808151636820
H	-1.74709662080228	4.57374128082073	3.09306768761694
H	1.01966026177244	-4.81611903772916	-3.07153785170937
H	-3.18524987264723	-4.26498864831529	2.59849275945940
H	4.49337412775030	2.18821764853474	-0.63832975977165
H	-0.16698286893222	2.17597123388768	-2.22322969679819
H	1.41505869620837	3.84438434640437	-3.17800506923866
H	4.95877244179508	0.61483819536914	0.64797893655746
H	3.27951926306908	-2.46154634008747	3.17491564605721
H	-4.02309738749358	2.95989914985111	-0.56701604429001
H	-1.68359741603231	-0.90245526090556	-2.54640779954163
H	-3.79932406412967	-0.11336798499584	-3.59954101458308
H	-3.01450382485221	3.79731600000692	1.09159935675990
H	0.32437884857039	3.30567845519114	3.79286807032726
H	1.02038111584434	1.32659336319446	2.46224940987045
H	1.04713227674785	-1.93261807489967	2.20990748309329
H	-1.88824625470388	-0.15052997643134	2.54494828798814
H	-3.24266864220201	-1.95131250124313	3.60696023293392
H	-0.46438563252298	-4.83993859087078	-1.06747527274353
H	2.05906092656712	-2.62751068774669	-3.79291484969394

H	1.56530697369178	-0.57903965399921	-2.47669378140354
H	-1.78794873607705	-4.66818645493949	0.57432756875551
